# EPC-exosomal miR-26a-5p improves airway remodeling in COPD by inhibiting ferroptosis of bronchial epithelial cells via PTGS2/PGE2 signaling pathway

**DOI:** 10.1038/s41598-023-33151-w

**Published:** 2023-04-14

**Authors:** Caihong Liu, Junjuan Lu, Ting Yuan, Lihua Xie, Li Zhang

**Affiliations:** 1 Department of Pulmonary and Critical Care Medicine, The Third Xiangya Hospital of Central South University, 138 Tongzipo Road, Yuelu District, Changsha, 410013 Hunan China; 2 Department of Nutriology, Second Xiangya Hospital, Central South University, Changsha, 410001 Hunan China

**Keywords:** Cell biology, Immunology, Diseases

## Abstract

We aimed to investigate whether exosomes (Exo) affected chronic obstructive pulmonary disease (COPD) by influencing ferroptosis of bronchial epithelial cells (BECs) and the mechanisms involved. Here we took the peripheral blood samples of normal subjects and COPD patients, extracted and identified endothelial progenitor cells (EPCs) and EPC-Exo. An animal model of COPD was established. Then human BECs were taken and treated with cigarette smoke extract (CSE) for 24 h to construct a COPD cell model. Next, we screened differentially expressed ferroptosis-related genes in COPD patients by bioinformatics. Bioinformatics predicted the miRNA targeting PTGS2. Then, the mechanism of action of miR-26a-5p and Exo-miR-26a-5p was investigated in vitro. We successfully isolated and identified EPC and Exo. In vitro, EPC alleviated CSE-induced ferroptosis in BECs by transporting Exo. In vivo, Exo alleviated cigarette smoke-induced ferroptosis and airway remodeling in mice. Through further validation, we found that CSE-induced ferroptosis promoted the epithelial-mesenchymal transition (EMT) of BECs. Bioinformatics analysis and validation showed that PTGS2/PGE2 pathway affected CSE-induced ferroptosis in BECs. Meanwhile, miR-26a-5p targeting PTGS2 affected CSE-induced ferroptosis in BECs. Additionally, we found that miR-26a-5p affected CSE-induced BECs EMT. Exo-miR-26a-5p alleviated CSE-induced ferroptosis and EMT. In conclusion, EPC-exosomal miR-26a-5p improved airway remodeling in COPD by inhibiting ferroptosis of BECs via the PTGS2/PGE2 pathway.

## Introduction

Chronic obstructive pulmonary disease (COPD) is one of the fourth leading causes of morbidity and mortality in the world^1^. Airway remodeling is one of its main pathological features^2^. Bronchial epithelial cells (BECs) are the first anatomical barrier to exposure to noxious gases and cigarette smoke (CS) that initiating airway remodeling in COPD^3^. Moreover, airway epithelial-mesenchymal transformation (EMT) is a highly plastic process by which epithelial cells transform into a mesenchymal phenotype after epithelial injury, resulting in airway wall thickening^4^. Ferroptosis is an iron-dependent form of regulated cell death characterized by massive lipid peroxide accumulation and an imbalance in the redox state of the cell^5^. In COPD, ferroptosis is an important mechanism of damage^6^. Studies have shown that CS could induce ferroptosis in BECs^7^. From this, we could speculate that ferroptosis of BECs might lead to the destruction of the epithelial barrier to a certain extent, trigger airway remodeling, and promote the development of COPD.

Extracellular vesicles, especially exosomes (Exo), are gaining increasing attention from pulmonologists as tools for understanding pathogenesis and diagnosing diseases^8^. Exo has been shown to inhibit cellular ferroptosis in various diseases. In a model of acute myocardial infarction, mesenchymal stem cells-Exo could alleviate myocardial injury by inhibiting ferroptosis^9^. Carcinoma-associated fibroblasts (CAFs) inhibited ferroptosis of gastric cancer cells by secreting Exo^10^. In a previous study, it was reported that endothelial progenitor cell (EPC) derived Exo was administered intratracheally to a mouse model of lipopolysaccharide (LPS)-induced acute lung injury, and EPC-Exo was found to reduce myeloperoxidase activity, lung injury score, pulmonary edema and LPS-induced acute lung injury^11^. Exo has emerged as a new mediator of intercellular communication, but its role in CS-induced COPD remains unclear.

Through bioinformatics analysis, we obtained the ferroptosis-related differential gene prostaglandin-endoperoxide synthase 2 (PTGS2), also known as cyclooxygenase-2 (COX-2). PTGS2 is the ferroptosis marker. COX-2 was significantly up-regulated in chronic and acute inflammation^12^. Studies have shown that cigarette smoke extract (CSE) could induce elevated PTGS2 expression in COPD^13–15^. It was found that activation of the COX-2/PGE2 pathway could abolish the protective effect of EPC-Exo miR-137 on ferroptosis in SH-SY5Y cells^16^. Meanwhile, the PTGS2/PGE2 pathway has a regulatory effect on EMT. In prostate cancer metastasis studies, it was found that EMT of prostate cancer cells could be affected by inhibiting COX-2/PGE2/STAT3 axis^17^. In cholangiocarcinoma, COX-2/PGE2 could influence EMT to promote cancer metastasis^18^. These studies suggest that the PTGS2/PGE2 signaling axis may have an important role in ferroptosis and EMT processes. However, the mechanism involved in the PTGS2/PGE2 signaling pathway in COPD remains unclear.

Therefore, in this study, we explored whether Exo and PTGS2/PGE2 signaling pathway affect COPD by influencing the ferroptosis of BECs by constructing a model of COPD. We found that EPC-Exo miR-26a-5p ameliorated airway remodeling in COPD by inhibiting ferroptosis of BECs via the PTGS2/PGE2 signaling pathway. This study could improve the understanding of the pathogenesis of COPD and provide new targets and strategies for treating COPD.

## Materials and methods

### Clinical samples

10 normal subjects and 10 COPD patients were from The Third Xiangya Hospital of Central South University, and we obtained their peripheral blood. This study was approved by The IRB of Third Xiangya Hospital, Central South University (NO. 2022-S129) and has been performed following the Declaration of Helsinki. The written informed consent has been obtained from each subject in this study.

### Extraction and identification of EPCs and EPCs-Exo

Peripheral blood samples from normal subjects and COPD patients were taken and added to heparin sodium (9 mL). Endothelial cells were extracted according to previous reports^19^. Cells from passages 3 to 5 were used in this study. CD146, CD34, and CD45 expressions were detected by flow cytometry (FCM, FACSCalibur) and BD FACSDiva 7.0 software (BD Biosciences). Additionally, EPCs-Exo were isolated and extracted using an Exo extraction kit (#60500, Norgen Biotek). The Exo morphology was detected by transmission electron microscope (TEM), and Exo markers CD9 (20597-1-AP, 1:1000, proteintech), TSG101 (14497-1-AP, 1:3000, proteintech), and CD63 (25682-1-AP, 1:1000, proteintech), and endoplasmic reticulum marker HSP90B1 (14700-1-AP, 1:2000, proteintech) were detected by western blot. The EPCs and their Exo extracted from normal subjects were labeled as: EPC and Exo, respectively.

### Establishment of COPD animal model

Forty C57BL/6 mice aged 6–8 weeks were randomly divided into the Control group (non-smoking control group, n = 10), Control + Exo group (n = 10), Model group (smoking group, n = 10) and M + Exo group (Model + Exo group, n = 10). In each experiment, age and sex were matched in each group. Among them, the mice exposed to the air served as the Control group. Mice in the Model group were exposed to CS^7^. Mice were exposed in a barrier facility using a systemic exposure system (SCIREQ “inexposure”). Mice were exposed to total suspended particulates at 200 mg/m^3^ weekly for 5 days for 6 months using cigarettes. Control + Exo group or M + Exo group was treated with the same treatment as the Control or Model group, and at the same time, 10 days before the last CS exposure, 15 μg/day EPC-Exo (labeled with PKH26 in advance) was intraperitoneally injected^20^. Control and Model groups were injected with the same amount of normal saline. After the last treatment, mouse bronchoalveolar lavage fluid (BALF) was collected. The mice were euthanized by cervical dislocation after intraperitoneal injection of pentobarbital (150 mg/kg) and whole lung tissues were collected. In vivo imaging showed the uptake of Exo in 0 d, 5 d, and 10 d animals. The experiments on animals were approved by The IRB of Third Xiangya Hospital, Central South University (NO. 2022-S129) and all methods were carried out in accordance with the ARRIVE guidelines and relevant regulations.

### Hematoxylin–eosin (HE) staining

Bronchial wall thickness was assessed under a light microscope. The slices were baked, dewaxed, and stained with hematoxylin and eosin. Then slices were dehydrated with gradient alcohol. After taking them out, they were placed in xylene, sealed with neutral gum, and observed under the microscope.

### Preparation of CSE

Approximately 30–50 mL of CS was drawn into the syringe and bubbled into sterile PBS in the 15 mL BD falcon tube. We made 10 mL of the solution in one cigarette. CSE solution was filtered (0.22 μm, Merck Millipore, SLGS033SS) to remove insoluble particles and designated as 100% CSE solution^7^.

### Cell culture and treatment

Human BECs were purchased from ATCC. BECs were treated with 5% CSE for 24 h to construct a COPD cell model^7^. They were divided into the Control group (PBS-treated BECs) and the CSE group (CSE-treated BECs). Then groups were further divided into the Control group (PBS-treated BECs), CSE group (CSE-treated BECs), and CSE + DFO group (100 µM desferrioxamine (DFO) was added to BECs 1 h before 5% CSE treatment). Next, PTGS2 was knocked down, and the groups were divided into the Control group, CSE group, CSE + NC group, and CSE + sh-PTGS2 group. Then miR-26a-5p was overexpressed and grouped into the Control group, CSE group, CSE + NC group, and CSE + miR-26a-5p mimics group. Additionally, PTGS2 and miR-26a-5p were overexpressed in CSE-treated BECs. The groups were divided into the NC group, miR-26a-5p mimics group, miR-26a-5p mimics + vector group, and miR-26a-5p mimics + PTGS2 mimics group. Finally, miR-26a-5p was knocked down and grouped into the CSE group (BECs treated with CSE), CSE + Exo group (Exo extracted from EPC), CSE + Exo-NC inhibitor group (Exo extracted from EPC transfected with NC inhibitor), and CSE + Exo-miR-26a-5p inhibitor group (Exo extracted from EPC transfected with miR-26a-5p inhibitor). All the groups are n = 3.

### Cell co-culture

First, cells were grouped into the CSE group (BECs treated with CSE), CSE + Normal EPC group (CSE-treated BECs were co-cultured with normal EPCs), and CSE + COPD EPC group (CSE-treated BECs were co-cultured with COPD EPCs). Next, before cell co-culture, EPCs were treated with exo production inhibitor 2.5 mM GW4869 (Sigma-Aldrich) or 0.005% DMSO for 3 h^21^. BECs were treated with 5% CSE for 24 h. Then the above cells were co-cultured for 48 h^22^ and grouped into the Control group (BECs treated with PBS), CSE group (BECs treated with CSE), EPC + DMSO group (EPCs treated with 0.005% DMSO were co-cultured with CSE-treated BECs), and EPC + GW4869 group (2.5 mM GW4869-treated EPCs were co-cultured with CSE-treated BECs). All the groups are n = 3.

### TEM

BECs were fixed to 2.5% glutaraldehyde. Then cells were put into PBS, fixed with 1% osmic acid for 1–2 h. All levels of ethanol were dehydrated, soaked, embedded in pure epoxy resin, and then baked in an oven at 40 °C for 12 h and at 60 °C for 48 h. Slices were stained with lead citrate, and BECs structure was observed by TEM.

### Cell counting kit-8 (CCK-8) assay

Cells were digested, counted, and seeded in a 96-well plate at a density of 5 × 10^3^ cells/well, with 100 μL per well. After culturing adherent cells, cells were treated as above for a corresponding time, and 10 μL/well of CCK8 was added to each well. After incubation at 37 °C and 5% CO_2_ for 4 h, the absorbance at 450 nm was analyzed by a microplate reader.

### TUNEL

Cell apoptosis was measured by the TUNEL apoptosis detection kit (40306ES50, Yeasen). Cells were fixed with 3% paraformaldehyde. After washing with PBS, 0.1% TritonX-100 and 0.1% sodium citrate solution were added and permeabilized at 4 °C for 5 min. Cells were washed again and treated with fluorescently labeled nucleotides dUTP and TdT for 60 min at 37 °C. TUNEL-positive cells were analyzed under a fluorescence microscope.

### Exo uptake assay

BECs were digested with 0.25% trypsin solution (containing 0.02% EDTA), and digestion was terminated by serum. BECs were centrifuged at 1000 rpm for 5 min. We discarded the supernatant, and added DMEM containing 10% FBS to resuspend cells. Cells were seeded in 12-well plates pre-placed with slides at 5 × 10^4^/mL and cultured overnight. 10% CSE intervened cells for 24 h. Exo was labeled with PKH26 dye (PKH26PCL, Sigma) and incubated with cells. The slices were taken out, fixed with 4% paraformaldehyde. 1 mL of 1 μg/mL DAPI was added, incubated at 37 °C for 10 min, and observed under the fluorescence microscope.

### Enzyme-linked immunosorbent assay (ELISA)

With MDA (#A003-1, Nanjing jiancheng Bioengineering Institute), SOD (#A001-3, Nanjing jiancheng Bioengineering Institute), TNF-α (#CSB-E04741m, CUSABIO), IL-1β (#CSB-E08054m, CUSABIO), HMGB1 (#CSB-E08225m, CUSABIO), IL-6 (#CSB-E04639m, CUSABIO), TGF-β (#CSB-E04726m, CUSABIO), and PGE2 (#CSB-E07965h, CUSABIO) quantitative ELISA kits, related factors were tested according to instructions. The DYNATECHMR7000 microplate reader was used to calculate the concentration of each indicator by forming the standard curve through the constant value provided.

### FCM

The content of catalytic Fe (II) was detected according to FerroFarRed kit (GC903-01, GORYO CHEMICAL). First, the medium was discarded, and cells were washed with PBS. 1 mM FerroFarRed stock solution was diluted into 5 μM working solution with serum-free medium, incubated with cells at 37 °C for 1 h. Then we discarded the dye solution, washed once with PBS, digested cells with 0.25% trypsin solution for 5 min, collected cells, and centrifuged cells at 500 g for 5 min. The supernatant was discarded, the pellet was resuspended in PBS, and the cell suspension was passed through a 40 μm nylon mesh to remove cell debris. SiRhoNox-1 was applied to measure the fluorescence signal at 633 nm (660/40).

ROS content was detected using the ROS detection kit (S0033S, Nanjing Jiancheng Biological Engineering Research Institute). Cells were harvested, and DCFH-DA was diluted 1:1000 in a serum-free medium. The released DCFH-DA was added to resuspend cells. An unstained sample was set simultaneously and incubated at 37 °C for 20 min. The cell pellet was collected by centrifugation, and cells were washed twice with a serum-free medium. Within 1 h, FCM was performed.

### Masson staining

Mouse bronchial samples were fixed in 4% paraformaldehyde for more than 24 h. Sections were deparaffinized, dropped nuclear staining solution, and stained. Sections were soaked in a weak alkaline solvent, added slurry dye, and stained for 2 min. The color separation solution was separated for about 30 s. The counterstaining solution was dropped, stained, sealed with neutral gum, and observed under the microscope.

### Quantitative real-time PCR (qRT-PCR)

Total RNA was extracted by the Trizol method. With a cDNA reverse transcription kit (#CW2569, Beijing ComWin Biotech, China), RNA was reverse transcribed into cDNAs and genes were tested by Ultra SYBR Mixture (#CW2601, Beijing ComWin Biotech, China). Using β-actin or U6 as internal reference, the gene level was calculated by the 2^-ΔΔCt^ method. The primer sequences are as follows: PTGS2-F: GTTCCACCCGCAGTACAGAA, PTGS2-R: AGGGCTTCAGCATAAAGCGT; HMOX1-F: CACACCCAGGCAGAGAATGCT, HMOX1-R: GGCTCTCCTTGTTGCGCTCA; MT1G-F: AAAGGGGCATCGGAGAAGTG, MT1G-R: GCAAAGGGGTCAAGATTGTAGC; miR-26-5P-F: TGGCCTCGTTCAAGTAATCCA, miR-26-5P-R: CCCCGTGCAAGTAACCAAGA; U6-F: CTCGCTTCGGCAGCACA, U6-R: AACGCTTCACGAATTTGCGT; β-actin-F: ACCCTGAAGTACCCCATCGAG, β-actin -R: AGCACAGCCTGGATAGCAAC.

### Western blot

Total protein was extracted by RIPA (P0013B, Beyotime). Following the instructions for the use of the BCA protein quantification kit, we determined the protein concentration. Proteins were separated by 10% SDS-PAGE electrophoresis. Electrotransfer transferred proteins to PVDF membranes. For primary antibodies, we used TfR (10084-2-AP, 1:1000, proteintech), GPX4 (Ab125066, 1:1000, abcam), FtL (10727-1-AP, 1:1000, proteintech), E-cadherin (20874-1-AP, 1:20,000, proteintech), Vimentin (10366-1-AP, 1:5000, proteintech), ZO-1 (21773-1-AP, 1:5000, proteintech), PTGS2 (27308-1-AP, 1:1000, proteintech), and β-actin (66009-1-Ig, 1:5000, proteintech). HRP-labeled secondary antibodies were then incubated. The membrane was immersed in ECL chemiluminescent solution (K-12045-D50, advansta) for luminescence visualization. β-actin acted as the internal reference.

### Immunofluorescence (IF)

IF was performed to detect the expression levels of EMT markers Vimentin and E-cadherin in BECs. The slices were removed, washed with PBS, fixed in 4% paraformaldehyde for 30 min, and rinsed 3 times with PBS for 5 min each. Then, the slices were permeabilized with 0.5% TritonX-100 for 30 min at 37 °C. After PBS rinsing, 5% BSA was closed at 37 °C for 1 h. Vimentin (10366-1-AP, 1:50, proteintech) and E-cadherin (20874-1-AP, 1:50, proteintech) primary antibodies were incubated overnight at 4 °C. Diluted fluorescent secondary antibody CoraLite488-conjugated Affinipure Goat Anti-RabbitIgG(H + L) (SA00013-2, 1:100, Proteintech) was then incubated at 37 °C for 90 min. DAPI working solution was applied to stain nucleus at 37 °C for 10 min and rinsed in PBS for 5 min. The slices were sealed with buffered glycerol and observed under a fluorescent microscope.

### Bioinformatics prediction

Raw datasets of GSE38974 (training set) was downloaded in the GEO database, converted to expression matrix, and grouping information was obtained. There were 23 cases of COPD samples and 9 healthy controls. The expression matrix was processed using the R language limma package, and differential expression analysis was applied to get differentially expressed genes (DEGs). Up- and down-regulated genes with statistical significance were screened according to P value and logFC (mRNA screening criteria: P value < 0.05 and |logFC|> 1.0), and volcano maps of DEGs were drawn. Then 60 ferroptosis-related genes recorded in the literature were obtained, and the intersection of DEGs and ferroptosis-related genes was screened to get the Venn diagram. Based on the screening results in the previous step, a cluster heat map was drawn to show expression patterns in the training set of differentially expressed ferroptosis genes.

### Dual-luciferase assay

miR-26a-5p and PTGS2 binding sites were predicted by bioinformatics and verified by dual-luciferase assay kit (E1910, Promega) and grouped as the wt-PTGS2 + NC group, wt-PTGS2 + miR-26a-5p mimics group, Mut-PTGS2 + NC group, and Mut-PTGS2 + miR-26a-5p mimics group. Finally, luciferase activity was measured by GloMax 20/20 chemiluminescence detector (Promega).

### Complying with ethics of experimentation

All work was conducted with the formal approval of the local human subject or animal care committees (The Third Xiangya Hospital of Central South University, China) (No. 2022-S129), and that clinical trials have been registered as legislation requires.

### Statistical analysis

Graphpad Prism 8.0 software was applied for statistical analysis. Measurement data were expressed as mean ± standard deviation, and unpaired t-test was utilized between the two groups that fit the normal distribution. The significance of three or more groups was compared by one-way ANOVA. Tukey was adopted for post-mortem testing. P < 0.05 indicated a statistically significant difference.

## Results

### EPC alleviated CSE-induced ferroptosis in BECs by transporting Exo

**Figure 1:**
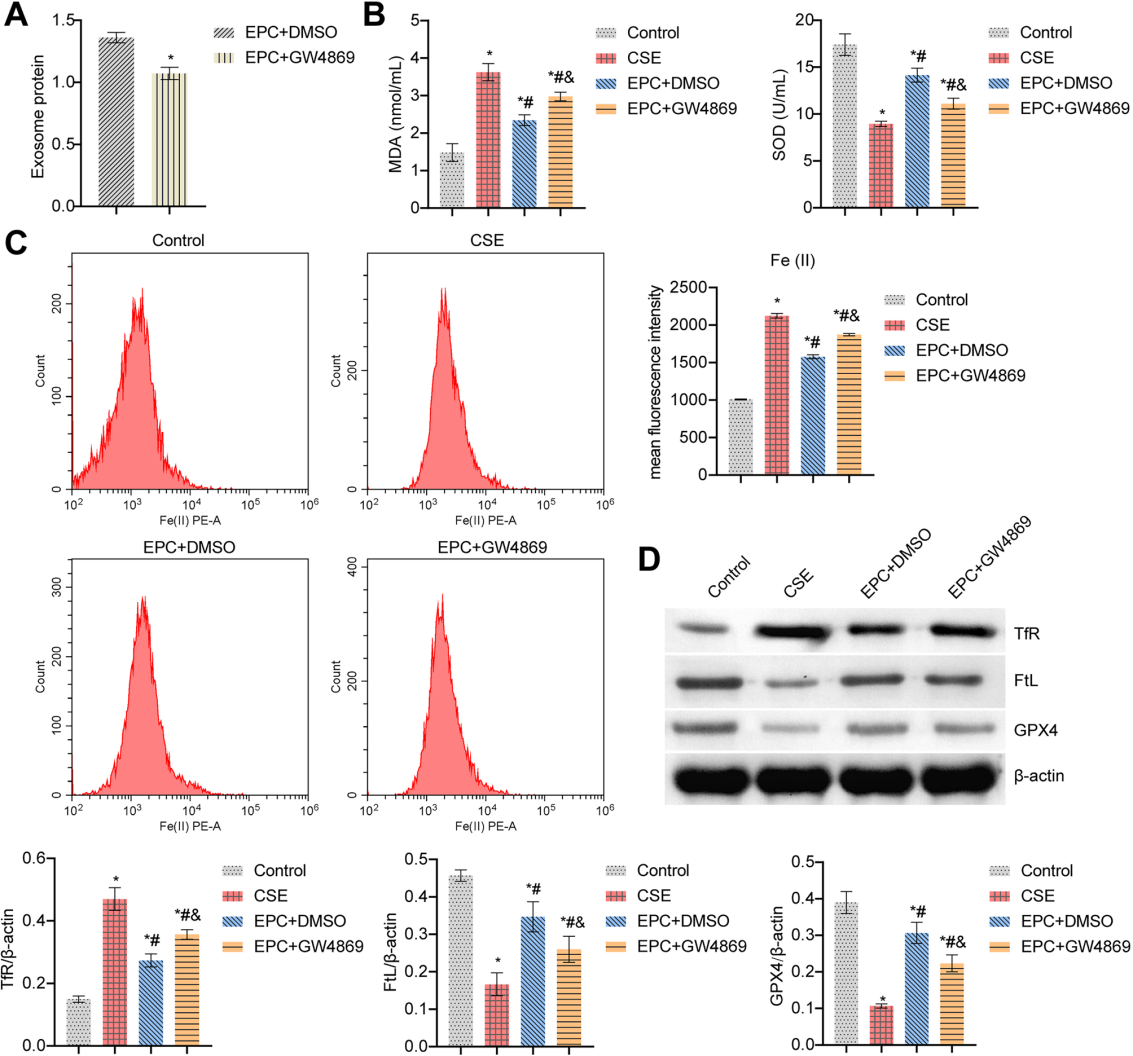
EPC alleviated CSE-induced ferroptosis in BECs by transporting Exo. (**A**) The concentration of Exo in the cell supernatant was evaluated by ELISA. *P < 0.05 vs. EPC + DMSO. (**B**) MDA and SOD levels in the cell supernatant were determined by ELISA. (**C**) The relative content of catalytic Fe (II) in BECs was detected by FCM. (**D**) Western blot detection of ferroptosis markers TfR, FtL, and GPX4 expressions in BECs. *P < 0.05 vs. Control, #P < 0.05 vs. CSE, &P < 0.05 vs. EPC + DMSO. The data were presented in the form of mean ± standard deviation. n = 3.

First, we characterized the extracted EPC and EPC-Exo. As shown in Fig. S1A, endothelial markers (CD146) were positive, CD34 was negative, and myeloid cell marker CD45 was negative, indicating successful EPC extraction. In addition, TEM revealed that Exo was oval vesicle-like (Fig. S1B). Compared with EPC, Exo markers (CD9, CD63, TSG101) were expressed in Exo, while endoplasmic reticulum marker (HSP90B1) was not expressed in Exo (Fig. S1C). These results showed that we had successfully isolated and identified Exo. Next, we processed the EPC. Compared with the EPC + DMSO group, Exo concentration in the EPC + GW4869 group was decreased (Fig. 1A). In addition, dense and shrunken mitochondria were evident in CSE-treated BECs compared with the Control group (Fig. S1D). This suggested that CSE induced ferroptosis in BECs. After CSE-treated BECs were co-cultured with normal EPCs or COPD EPCs, we found that the proliferation was increased and apoptosis was decreased in the CSE + Normal EPC and CSE + COPD EPC groups compared with the CSE group. Moreover, the CSE + COPD EPC group showed increased proliferation and a more pronounced decrease in apoptosis than CSE + Normal EPC (Fig. S1E,F). The CSE group had a higher MDA level and a lower SOD level than the Control group. The EPC + DMSO group had a lower MDA level and a higher SOD level than the CSE group. After adding GW4869, these indicators were reversed (Fig. 1B). In addition, CSE treatment increased Fe (II) and TfR levels and decreased FtL and GPX4 levels. Compared with the CSE group, the Fe (II) and TfR levels in the EPC + DMSO group were decreased, while FtL and GPX4 levels were increased. After adding GW4869, these indicators were reversed (Fig. 1C,D). These results suggested that EPC alleviated CSE-induced ferroptosis in BECs by transporting Exo.

### Exo alleviated ferroptosis induced by CS in vivo

**Figure 2:**
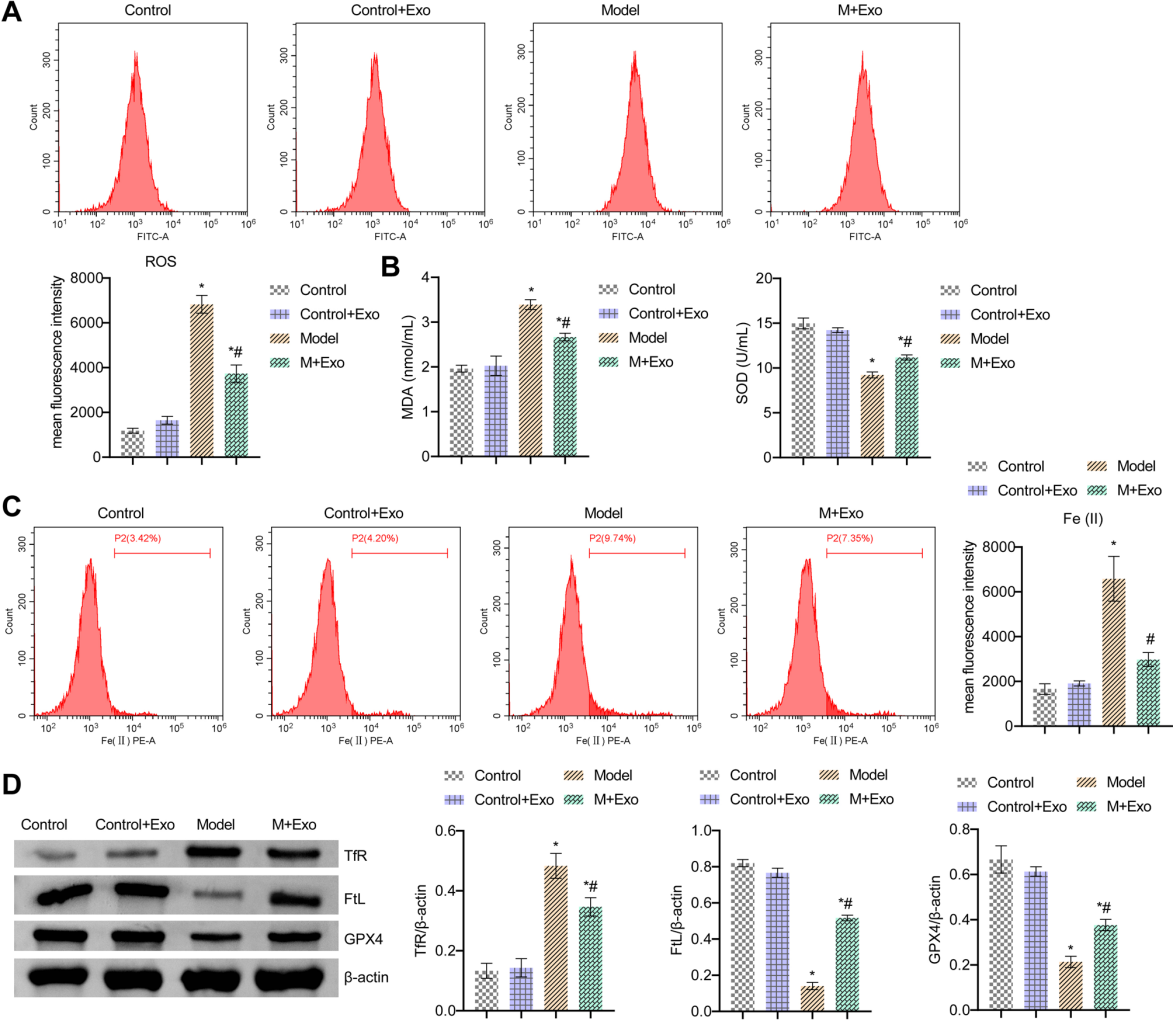
Exo alleviated ferroptosis induced by CS in vivo. (**A**) FCM detection of ROS content in lung tissue. (**B**) ELISA detection of MDA and SOD in lung tissue. (**C**) The relative content of catalytic Fe (II) in lung tissue was detected by FCM. (**D**) The relative expression of ferroptosis markers TfR, FtL, and GPX4 in lung tissue was evaluated by western blot. *P < 0.05 vs. Control, #P < 0.05 vs. Model. The data were presented in the form of mean ± standard deviation. n = 5 mice/group.

Next, we explored the role of Exo in vivo. The detection of oxidative stress markers in lung tissue showed that ROS and MDA levels in the Model group were facilitated, and SOD level was repressed. After using Exo, ROS and MDA levels decreased, but SOD levels increased in the M + Exo group (Fig. 2A,B). In addition, the levels of Fe (II) and TfR in the Model group were facilitated, while FtL and GPX4 levels were suppressed. After using Exo, the levels of Fe (II) and TfR decreased, while the levels of FtL and GPX4 increased in the M + Exo group (Fig. 2C,D). These results indicated Exo alleviated ferroptosis induced by CS in vivo.

### Exo alleviated CS-induced airway remodeling in mice

**Figure 3:**
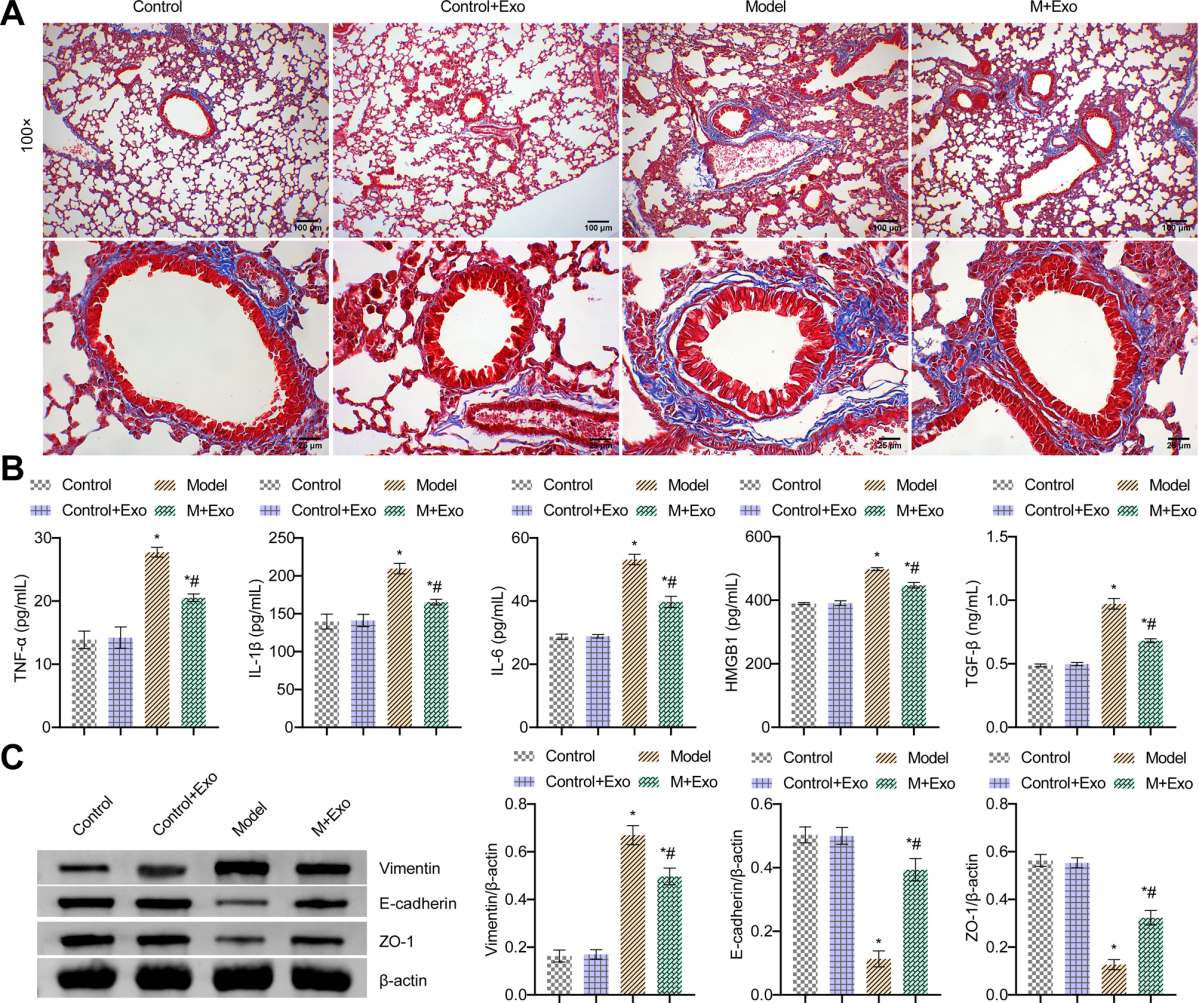
Exo alleviated CS-induced airway remodeling in mice. (**A**) Masson staining of the bronchus. (**B**) Expression of TNF-α, IL-1β, IL-6, HMGB1 and TGF-β in BALF were determined by ELISA. (**C**) Western blot detection of Vimentin, E-cadherin, and ZO-1 expressions. *P < 0.05 vs. Control, #P < 0.05 vs. Model. The data were presented in the form of mean ± standard deviation. n = 5 mice/group.

Masson staining showed increased CS exposure, thickening of the small airways and collagen accumulation in mice. The airway was relieved after using Exo in the M + Exo group (Fig. 3A). TNF-α, IL-1β, IL-6, HMGB1, and TGF-β levels in BALF were all accelerated in the Model group, but alleviated after using Exo in the M + Exo group (Fig. 3B). In addition, the levels of Vimentin increased in the Model group, and the levels decreased after Exo was used in the M + Exo group. The E-cadherin and ZO-1 expressions were opposite (Fig. 3C). These results suggested that Exo alleviated CS-induced airway remodeling in mice.

### CSE-induced ferroptosis promoted oxidative stress and EMT in BECs

**Figure 4:**
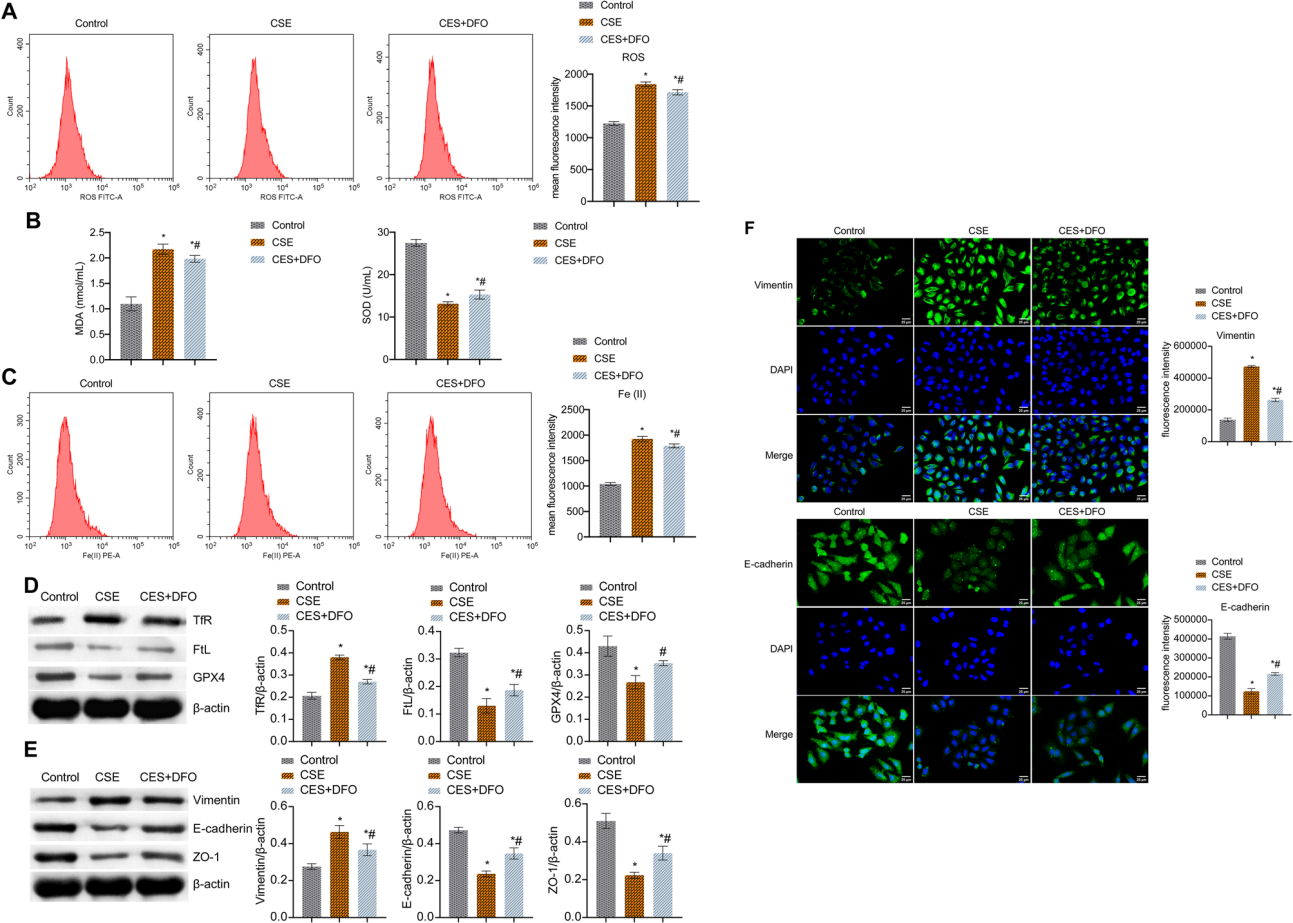
CSE-induced Ferroptosis promoted oxidative stress and EMT in BECs. (**A**) FCM detection of ROS content. (**B**) MDA and SOD levels. (**C**) The content of catalytic Fe (II) was detected by FCM. (**D**) The expression of ferroptosis markers TfR, FtL, and GPX4 was utilized by western blot. (**E**) The protein expression of EMT markers Vimentin, E-cadherin, and ZO-1. (**F**) IF staining of EMT markers Vimentin and E-cadherin. *P < 0.05 vs. Control, #P < 0.05 vs. CSE. The data were presented in the form of mean ± standard deviation. n = 3.

Next, we explored the mechanism of ferroptosis in BECs. Firstly, we examined markers of oxidative stress. In the CSE group, ROS and MDA levels were facilitated, but SOD levels were repressed. After adding DFO, ROS and MDA levels were reduced, but SOD levels were elevated (Fig. 4A,B). Moreover, Fe (II) and TfR levels in the CSE group were facilitated, while FtL and GPX4 levels were repressed. After adding DFO, Fe (II) and TfR levels were repressed, while FtL and GPX4 levels were facilitated (Fig. 4C,D). Finally, we examined markers of EMT. Vimentin level was increased in the CSE group compared with the control group, but decreased after adding DFO. The ZO-1 and E-cadherin expressions were opposite (Fig. 4E,F). These results indicated that CSE-induced ferroptosis promoted BECs oxidative stress and EMT.

### PTGS2/PGE2 signaling pathway affected CSE-induced ferroptosis in BECs

**Figure 5:**
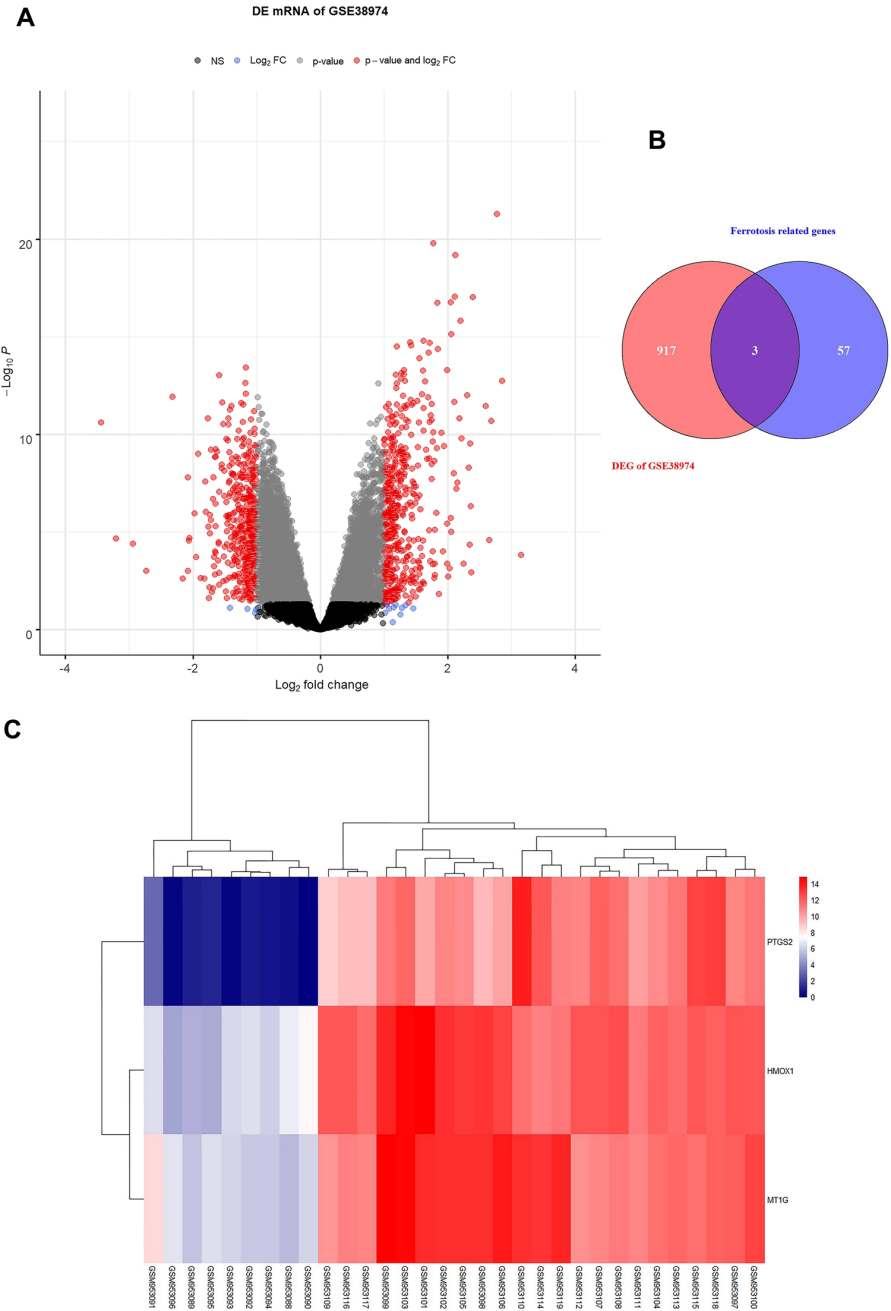

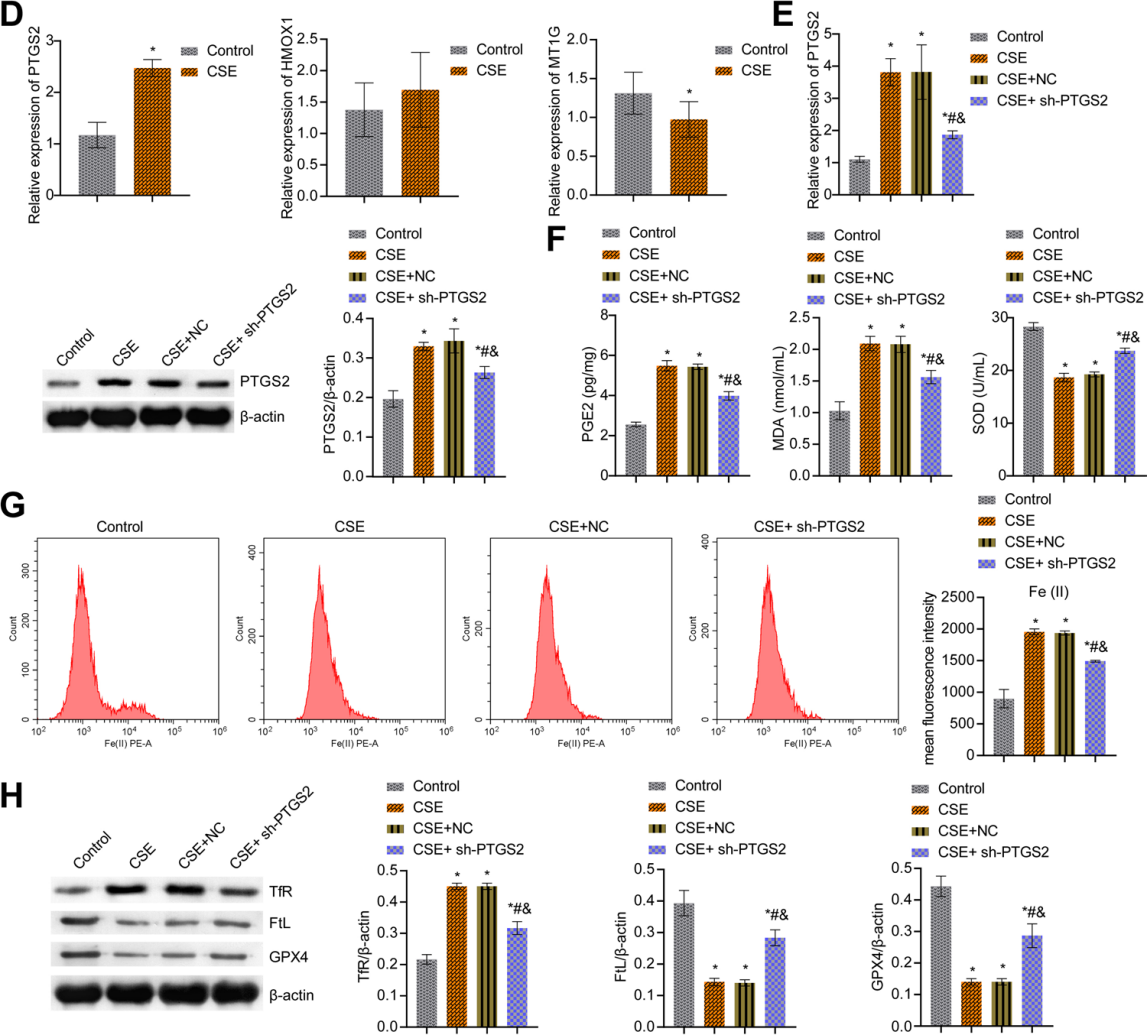
PTGS2/PGE2 signaling pathway affected CSE-induced ferroptosis in BECs. (**A**) Screening of DEGs. Red dots are differentially expressed mRNAs, and blue dots are stable genes. (**B**) Venn diagrams of DEGs and ferroptosis-related genes. (**C**) The cluster heatmap visualization of ferroptosis-related differential genes (drawn with R language). (**D**) qRT-PCR verification of PTGS2, HMOX1, and MT1G expression. (**E**) PTGS2 expression was determined by qRT-PCR and western blot. (**F**) ELISA detection of PGE2, and lipid peroxidation MDA and SOD. (**G**) The content of catalytic Fe (II) was detected by FCM. (**H**) The protein expression of ferroptosis markers TfR, FtL, and GPX4. *P < 0.05 vs. Control, #P < 0.05 vs. CSE, &P < 0.05 vs. CSE + NC. The data were presented in the form of mean ± standard deviation. n = 3.

Next, we screened differential genes in COPD patients by bioinformatics. As shown in Fig. 5A, a total of 920 DEGs (483 up-regulated genes and 437 down-regulated genes) were screened in the original expression matrix of GSE38974. Among them, the mRNA screening criteria were P value < 0.05 and |logFC|> 1.0. In addition, 60 ferroptosis-related genes recorded in the literature were obtained to screen the intersection of DEG and ferroptosis-related genes. The Venn diagram showed a total of 3 intersections of the GSE38974 training set with ferroptosis-related genes. That was, three ferroptosis-related genes (PTGS2, HMOX1, and MT1G) were significantly differentially expressed in DEGs (Fig. 5B). Thus, the clustering heat map was drawn to demonstrate the expression patterns in the training set of differentially expressed ferroptosis-related genes (PTGS2, HMOX1, and MT1G) based on the screening results of the Venn diagram. The results showed that the expression of ferroptosis-related genes (PTGS2, HMOX1, and MT1G) was upregulated in COPD compared with healthy controls (Fig. 5C). Next, we performed validation in cells. Among them, PTGS2 was highly expressed after CSE treatment, the difference was significant, and it was consistent with the predicted results of bioinformatics, so PTGS2 was selected for subsequent research (Fig. 5D). Next, we knocked down PTGS2. Figure 5E showed that PTGS2 was highly expressed after CSE treatment, and both mRNA and protein levels of PTGS2 decreased after interfering with PTGS2. PGE2 and MDA levels in the CSE group were increased, while SOD levels were decreased. After interfering with PTGS2, PGE2 and MDA levels were decreased, but SOD levels were facilitated (Fig. 5F). In addition, Fe(II) and TfR levels in the CSE group were facilitated, while FtL and GPX4 levels were reduced. After interfering with PTGS2, Fe (II) and TfR levels were reduced, and FtL and GPX4 levels were repressed (Fig. 5G,H). These results revealed that the PTGS2/PGE2 signaling pathway affected CSE-induced ferroptosis in BECs.

### miR-26a-5p targeting PTGS2 affected CSE-induced ferroptosis in BECs

**Figure 6:**
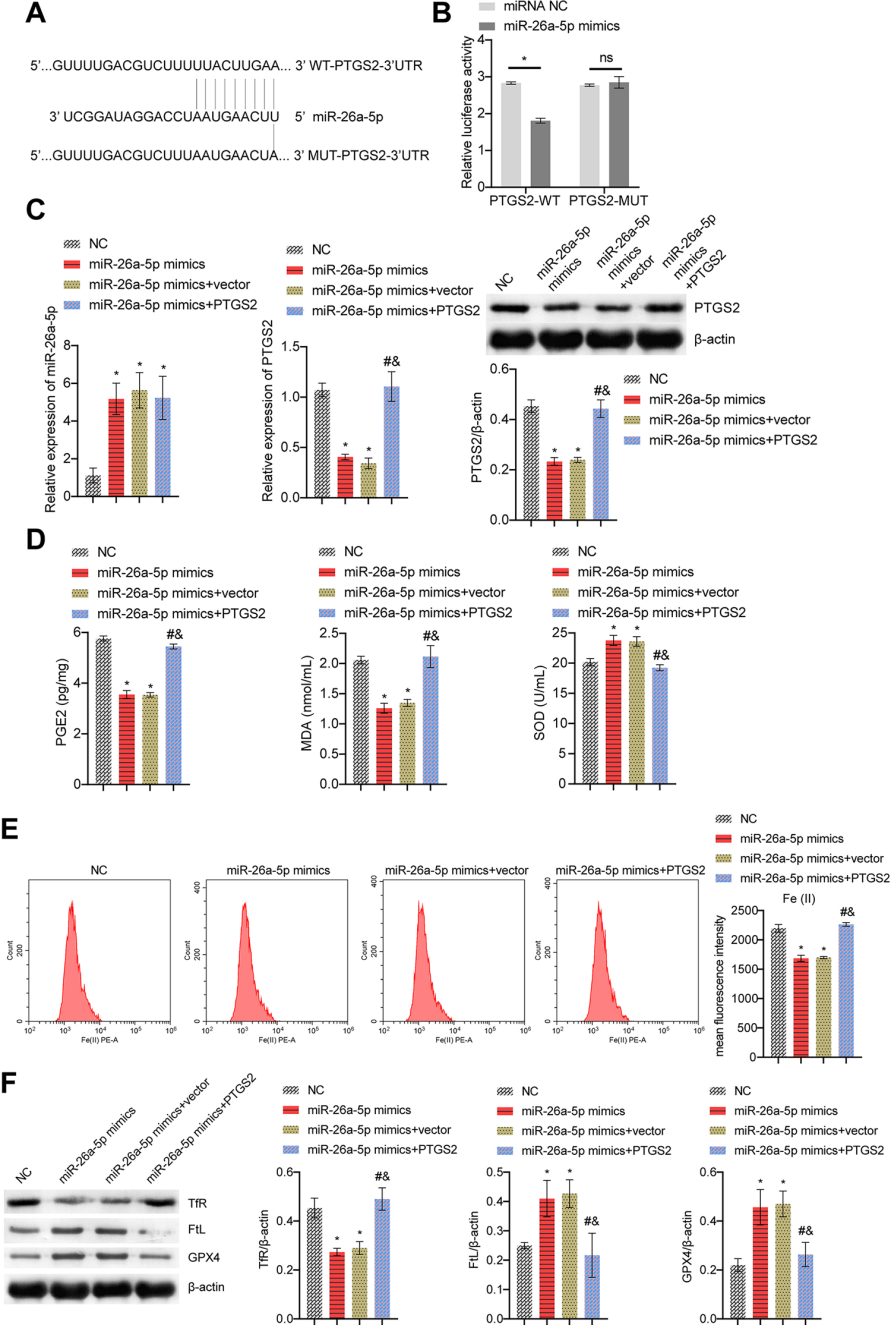
miR-26a-5p targeting PTGS2 affected CSE-induced ferroptosis in BECs. (**A**) Targeting sequences of miR-26a-5p and PTGS2. (**B**) Dual-luciferase verification of miR-26a-5p and PTGS2 relationship. (**C**) miR-26a-5p and PTGS2 mRNA expression, and PTGS2 protein expression. (**D**) ELISA detection of PGE2, lipid peroxidation MDA and SOD. (**E**) The content of catalytic Fe (II) was evaluated by FCM. (**F**) The protein expression of ferroptosis markers TfR, FtL, and GPX4. *P < 0.05 vs. NC, #P < 0.05 vs. miR-26a-5p mimics, &P < 0.05 vs. miR-26a-5p mimics + vector. The data were presented in the form of mean ± standard deviation. n = 3.

Firstly, Fig. 6A showed targeting sequences of miR-26a-5p and PTGS2. Dual-luciferase further verified the relationship between miR-26a-5p and PTGS2 (Fig. 6B). We then overexpressed miR-26a-5p and PTGS2. miR-26a-5p expression was increased and PTGS2 expression was decreased in the miR-26a-5p mimics group than the NC group. After overexpression of PTGS2, PTGS2 expression was increased (Fig. 6C). Furthermore, the miR-26a-5p mimics group decreased PGE2 and MDA levels, and facilitated SOD levels. After overexpression of PTGS2, PGE2 and MDA levels were facilitated, but SOD levels were suppressed (Fig. 6D). At the same time, after overexpression of miR-26a-5p, the levels of Fe(II) and TfR were decreased, while the levels of FtL and GPX4 were increased. After overexpression of PTGS2, Fe(II) and TfR levels were increased, and the levels of FtL and GPX4 were decreased (Fig. 6E,F). These results demonstrated miR-26a-5p targeting PTGS2 affected CSE-induced ferroptosis in BECs.

### miR-26a-5p affected CSE-induced BECs EMT

**Figure 7:**
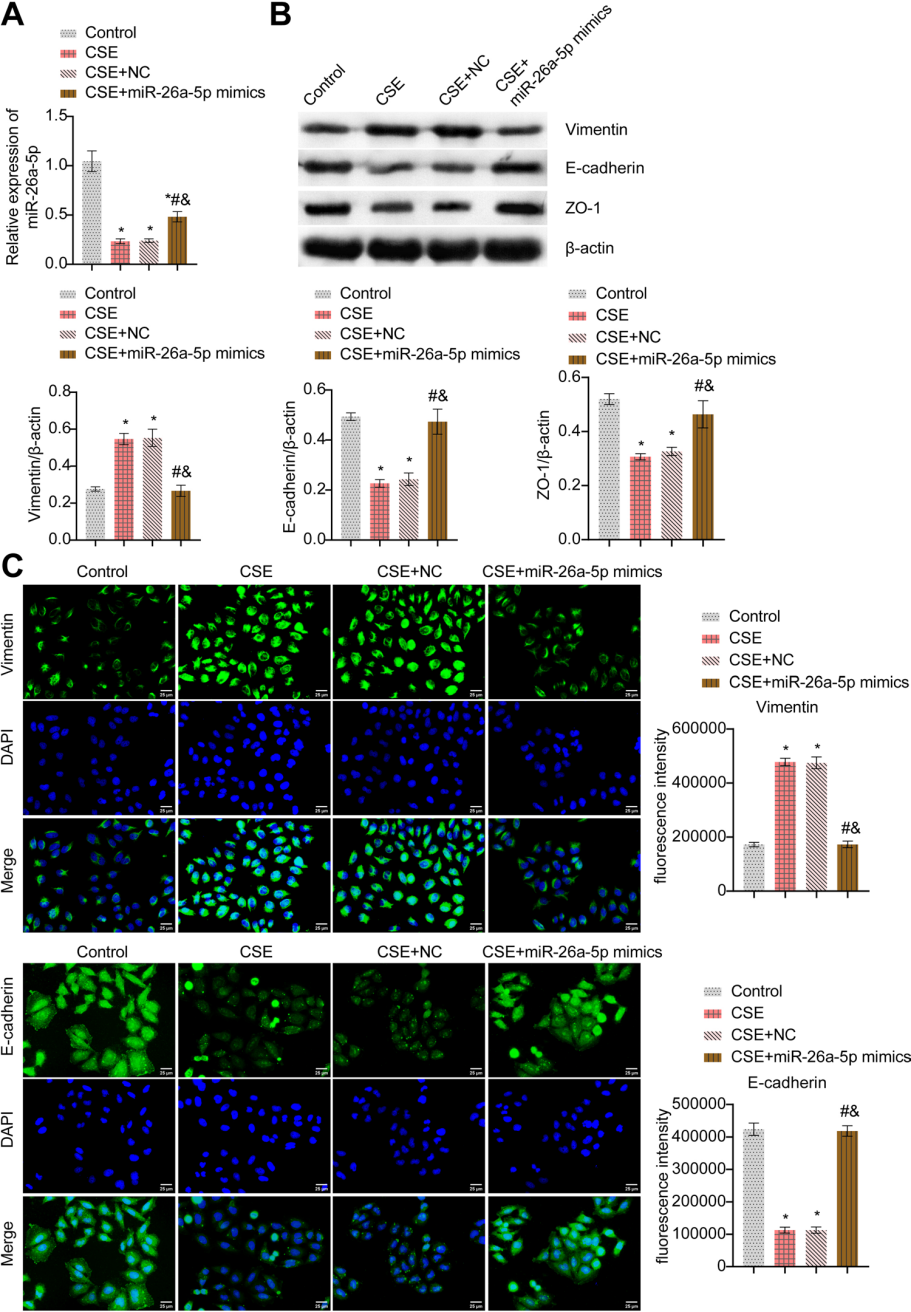
miR-26a-5p affected CSE-induced BECs EMT. (**A**) miR-26a-5p mRNA expression. (**B**) Western blot detection of EMT markers Vimentin, E-cadherin, and ZO-1. (**C**) IF staining of EMT markers Vimentin and E-cadherin. *P < 0.05 vs. Control, #P < 0.05 vs. CSE, &P < 0.05 vs. CSE + NC. The data were presented in the form of mean ± standard deviation. n = 3.

Next, we further explored the mechanism of action of miR-26a-5p. miR-26a-5p expression was repressed after CSE treatment. After overexpression of miR-26a-5p, miR-26a-5p expression was increased. This indicated miR-26a-5p was successfully overexpressed (Fig. 7A). Moreover, compared with the control group, Vimentin level was facilitated in the CSE group, but Vimentin level was suppressed after overexpression of miR-26a-5p. The E-cadherin and ZO-1 expressions were the opposite (Fig. 7B,C). In general, miR-26a-5p affected CSE-induced BECs EMT.

### Exo-miR-26a-5p alleviated CSE-induced ferroptosis and EMT

**Figure 8:**
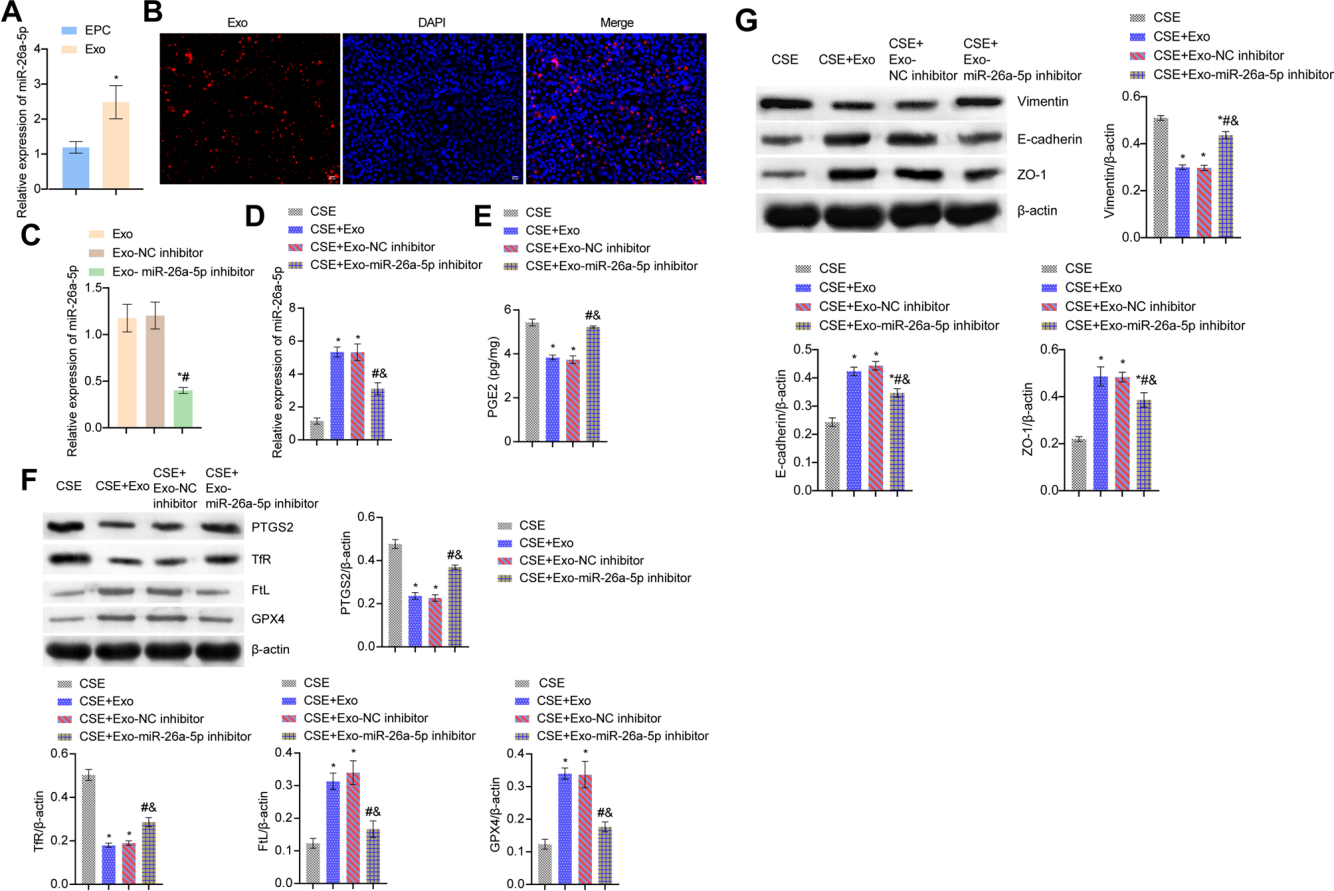
Exo-miR-26a-5p alleviated CSE-induced ferroptosis and EMT. (**A**) qRT-PCR verification of miR-26a-5p expression. *P < 0.05 vs. EPC. (**B**) Exo uptake assay. (**C**) miR-26a-5p mRNA expression. *P < 0.05 vs. Exo, #P < 0.05 vs. Exo-NC inhibitor. (**D**) miR-26a-5p mRNA expression. (**E**) ELISA detection of PGE2 expression. (**F**) The expressions of PTGS2 and ferroptosis markers TfR, FtL, and GPX4 were detected by western blot. G. EMT markers Vimentin, E-cadherin, and ZO-1 protein expression of BECs. *P < 0.05 vs. CSE, #P < 0.05 vs. CSE + Exo, &P < 0.05 vs. CSE + Exo-NC inhibitor. The data were presented in the form of mean ± standard deviation. n = 3.

We further found that miR-26a-5p was more highly expressed in the Exo group than the EPC group (Fig. 8A). Figure 8B showed Exo uptake experiment, that was, the successful uptake of miR-26a-5p by Exo. Next, we interfered with miR-26a-5p. miR-26a-5p expression in the Exo-miR-26a-5p inhibitor group was more repressed than the Exo-NC inhibitor group, suggesting that miR-26a-5p was successfully interfered (Fig. 8C). miR-26a-5p expression in the CSE + Exo group was increased than the CSE group. After interfering with miR-26a-5p, miR-26a-5p expression was alleviated (Fig. 8D). Under the treatment of CSE, PGE2 expression was alleviated after adding Exo, and PGE2 expression was accelerated after interfering with miR-26a-5p (Fig. 8E). In addition, under the treatment of CSE, after adding Exo, PTGS2 and TfR levels were alleviated, and FtL and GPX4 levels were accelerated. After interfering with miR-26a-5p, PTGS2 and TfR levels were accelerated, and FtL and GPX4 levels were reduced (Fig. 8F). Finally, we examined markers of EMT. Under CSE treatment, after adding Exo, Vimentin level was decreased, and after interfering with miR-26a-5p, Vimentin level was increased. The ZO-1 and E-cadherin expressions were the opposite (Fig. 8G). These results revealed that Exo-miR-26a-5p alleviated CSE-induced ferroptosis and EMT.

## Discussion

COPD is characterized by persistent respiratory symptoms and airflow limitation. CS causes epithelial cell death, leading to emphysema. Furthermore, CS induces iron accumulation in mitochondria and cytoplasm, leading to programmed cell death^23^. In this research, an animal model of COPD was established by exposure to CS, and BECs were treated with CSE to construct a COPD cell model to explore whether Exo affected COPD by influencing BEC ferroptosis and the mechanisms involved. Our results demonstrated that EPC-Exosomal miR-26a-5p improved airway remodeling in COPD by inhibiting ferroptosis of BEC via PTGS2/PGE2 signaling pathway. There is no report on the mechanism of EPC-Exosomal miR-26a-5p in COPD through PTGS2/PGE2 signaling pathway, which is also the innovation of this study.

Smoking is the most common risk factor for COPD^24^. The gas mixture produced by smoking has been shown to contain approximately 4500 constituents that are thought to be major factors driving lung disease pathogenesis and progression^25^. Inhalation of CS causes inflammatory cell infiltration into the mucosa, submucosa, and glandular tissues, resulting in matrix destruction, insufficient blood supply, and epithelial cell death. Ferroptosis is a necrotic form of regulated cell death characterized by the accumulation of lipid ROS through a ferrous ion-dependent Fenton reaction. Disruption of iron homeostasis leading to excessive oxidative stress has been implicated in COPD pathogenesis^26^. In this research, we found CSE-induced ferroptosis in BECs as well as CS-induced ferroptosis in vivo. Exos are produced by the inward budding of the membrane (endocytosis), followed by the formation of multivesicular bodies, which are released by exocytosis^27^. Studies have shown that exo released by different cells could serve as mediators for the exchange of information between different cells for carrying proteins, lipids and RNA molecules that promote cell-to-cell communication in both normal and diseased conditions^28^. Our study found that EPC alleviated CSE-induced ferroptosis in BECs by transporting Exo in vitro. In vivo, Exo alleviated CS-induced ferroptosis and airway remodeling in mice. This suggests that Exo plays an active regulatory role in COPD.

Airway remodeling is the structural change in the airway wall that occurs during repeated injury and repair. It occurs in patients with chronic inflammatory airway diseases, such as COPD^29^. The mechanism of airway remodeling is related to the EMT of the small airways in smokers and COPD patients^30^. In lung surface cells, CS induced inflammation and EMT^31^. We also found that CSE-induced ferroptosis promoted the EMT of BECs. Inducible COX-2 and inflammatory cytokines play vital roles in COPD inflammatory process^32^. Vascular endothelial cell apoptosis and COX-2 protein expression increased in COPD patients and CSE-induced cells. Interestingly, COX-2 may have a protective effect on the smoking-induced apoptosis of vascular endothelial cells^33^. COX-2 and its prostaglandin products, especially PGE2, are key factors in vitro models of exacerbation of infectivity^34^. The increase in PGE2 depends on the induction of COX-2^35^. PGE2 is a key component of the amplified and self-perpetuating cycle that induces senescence and inflammation in COPD fibroblasts^36^. Through bioinformatics analysis and validation, we showed that the PTGS2/PGE2 signaling pathway affected CSE-induced ferroptosis in BECs. This is the first time we have reported the study of the PTGS2/PGE2 signaling pathway and ferroptosis in COPD.

Studies have shown that miR-26a-5p is a potential regulator that might be involved in the pathogenesis of asthma^37^. Leidinger P et al. identified miRNAs associated with lung cancer and COPD isolation, including hsa-miR-26a-5p^38^. Through bioinformatics prediction and validation, we found that miR-26a-5p targeting PTGS2 affected CSE-induced ferroptosis in BECs. In addition, miR-26a-5p affected CSE-induced BECs EMT. With the further study of exo, more precise molecular mechanisms during cell-to-cell communication have been revealed; specifically, miRNAs are shuttled through exo^39^. Therefore, exosomal miRNAs are expected to be diagnostic biomarkers and therapeutic targets for COPD. Xu H et al. demonstrated that CS triggered the modification of exosomal components and recognized miR-21 from BECs as a mediator of myofibroblast differentiation by pVHL/HIF-1α pathway^21^. We found Exo-miR-26a-5p alleviated CSE-induced ferroptosis and EMT. This is the first time we report EPC-Exosomal miR-26a-5p role in COPD.

## Conclusions

Taken together, our findings suggested EPC-Exo-miR-26a-5p ameliorated airway remodeling in COPD by inhibiting ferroptosis in BECs via the PTGS2/PGE2 signaling pathway. Our research provides a theoretical basis for the pathogenesis of COPD, as well as new targets and strategies for the clinical treatment of COPD.

## Supplementary Information

Supplementary Figure S1.

Supplementary Information.

## Data Availability

Datasets of GSE38974 (https://www.ncbi.nlm.nih.gov/geo/query/acc.cgi?acc=GSE38974) and GSE103174 (https://www.ncbi.nlm.nih.gov/geo/query/acc.cgi?acc=GSE103174) analyzed during this study were downloaded in the GEO database. All data generated during this study are included in this article. Further enquiries can be directed to the corresponding author.
