# Supplementary material for: EPC-exosomal miR-26a-5p improves airway remodeling in COPD by inhibiting ferroptosis of bronchial epithelial cells via PTGS2/PGE2 signaling pathway
Source: Sci Rep. 2023 Apr 14;13:6126. doi: 10.1038/s41598-023-33151-w (PMC10104834; doi:10.1038/s41598-023-33151-w)
Supplement: Supplementary file 1 — Supplementary Figure S1. [file 41598_2023_33151_MOESM1_ESM.pdf]

Supplementary Materials

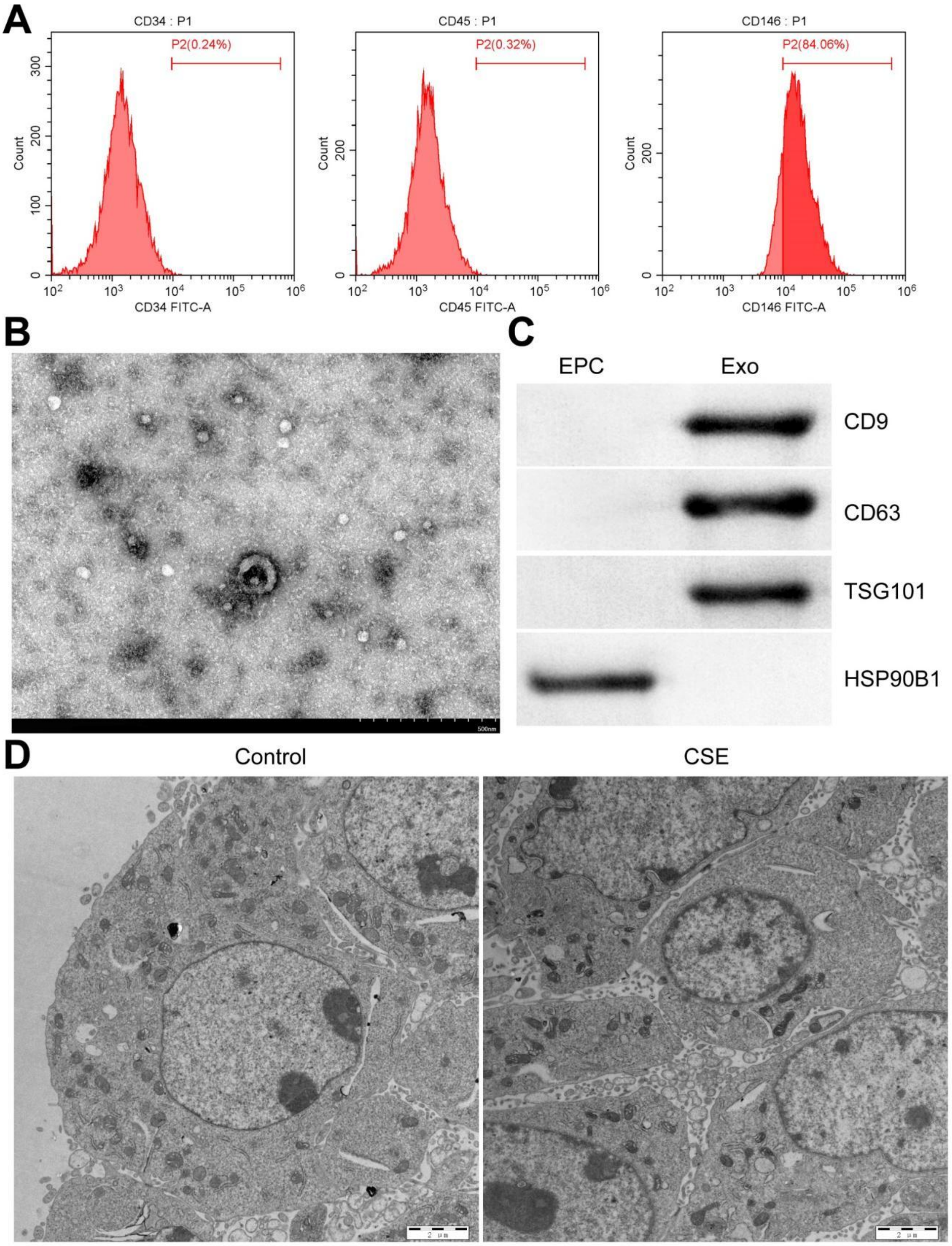

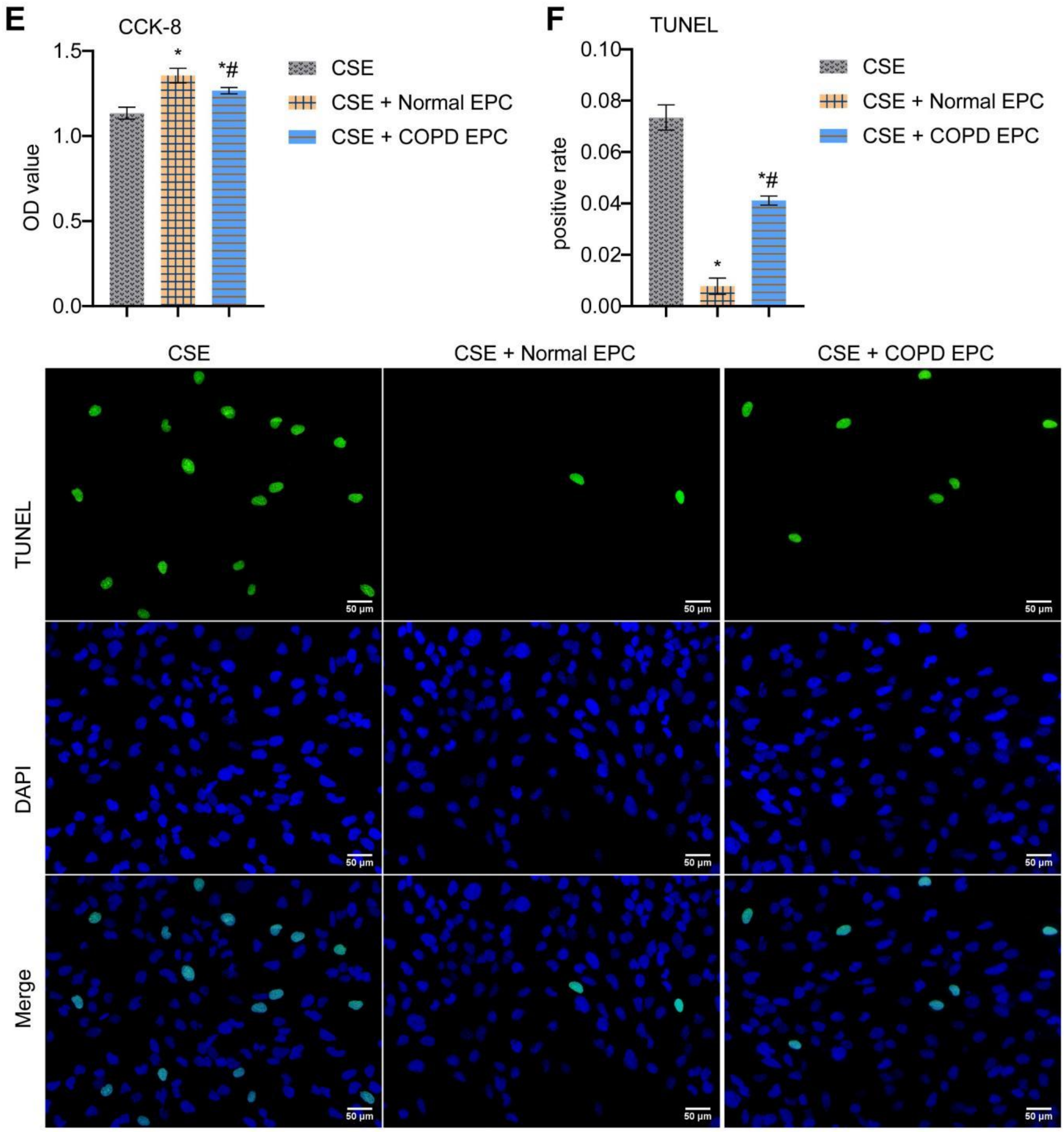

Figure S1. Identification of EPC and Exo. A. FCM identifies EPCs: CD146, CD34, and CD45. B. TEM of Exo morphology. C. Western blot detection of Exo expression: Exo markers (CD9, CD63, TSG101) and endoplasmic reticulum marker (HSP90B1). D. The mitochondrial status of BECs treated with CSE was observed by TEM. E. The proliferation activity of BECs was detected by CCK-8. F. The apoptosis of BECs was analysed by TUNEL staining. * P <0.05 vs CSE, # P <0.05 vs CSE+Normal EPC.
