# Supplementary material for: EPC-exosomal miR-26a-5p improves airway remodeling in COPD by inhibiting ferroptosis of bronchial epithelial cells via PTGS2/PGE2 signaling pathway
Source: Sci Rep. 2023 Apr 14;13:6126. doi: 10.1038/s41598-023-33151-w (PMC10104834; doi:10.1038/s41598-023-33151-w)
Supplement: Supplementary file 2 — Supplementary Information. [file 41598_2023_33151_MOESM2_ESM.pdf]

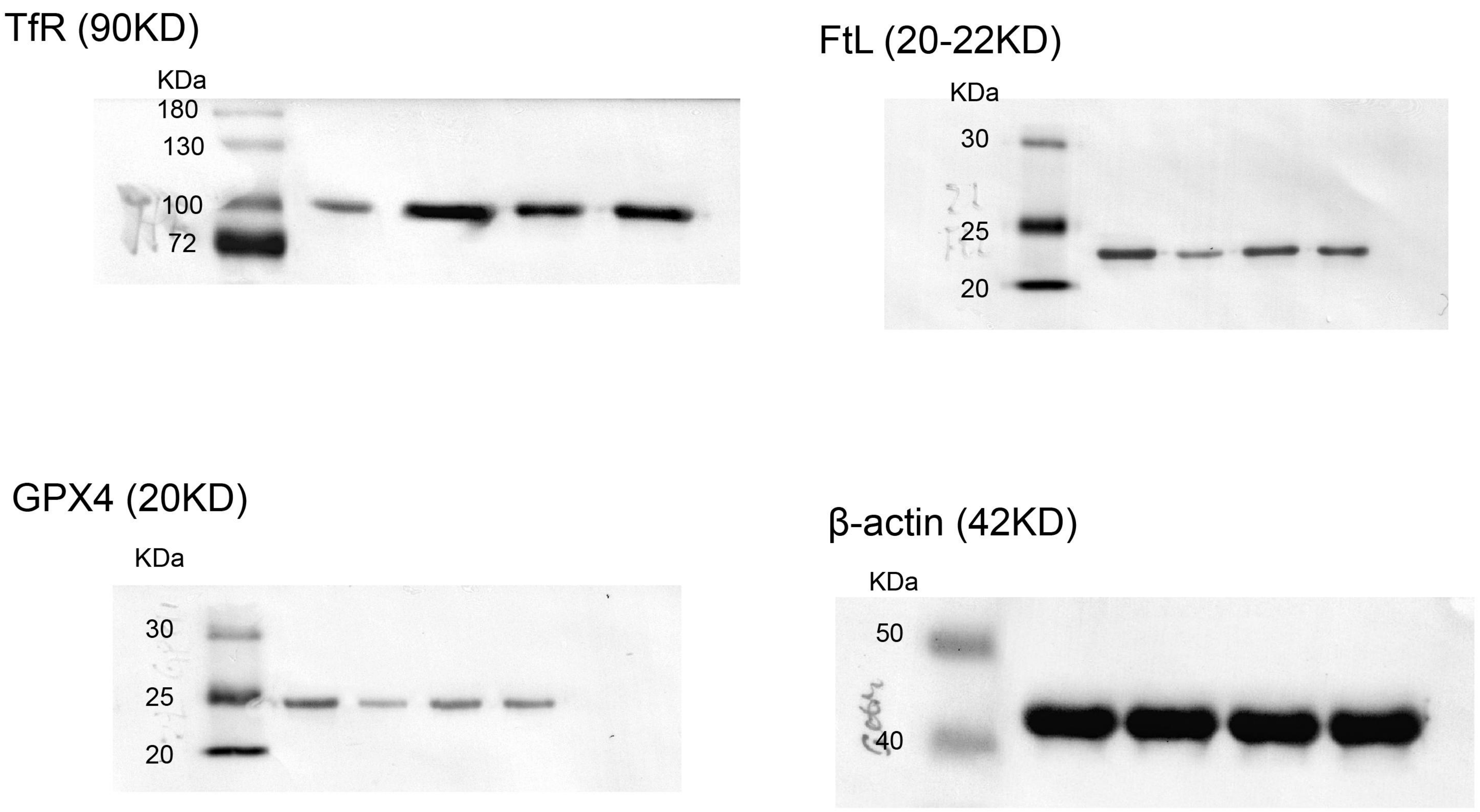

Original Image for Fig 1D

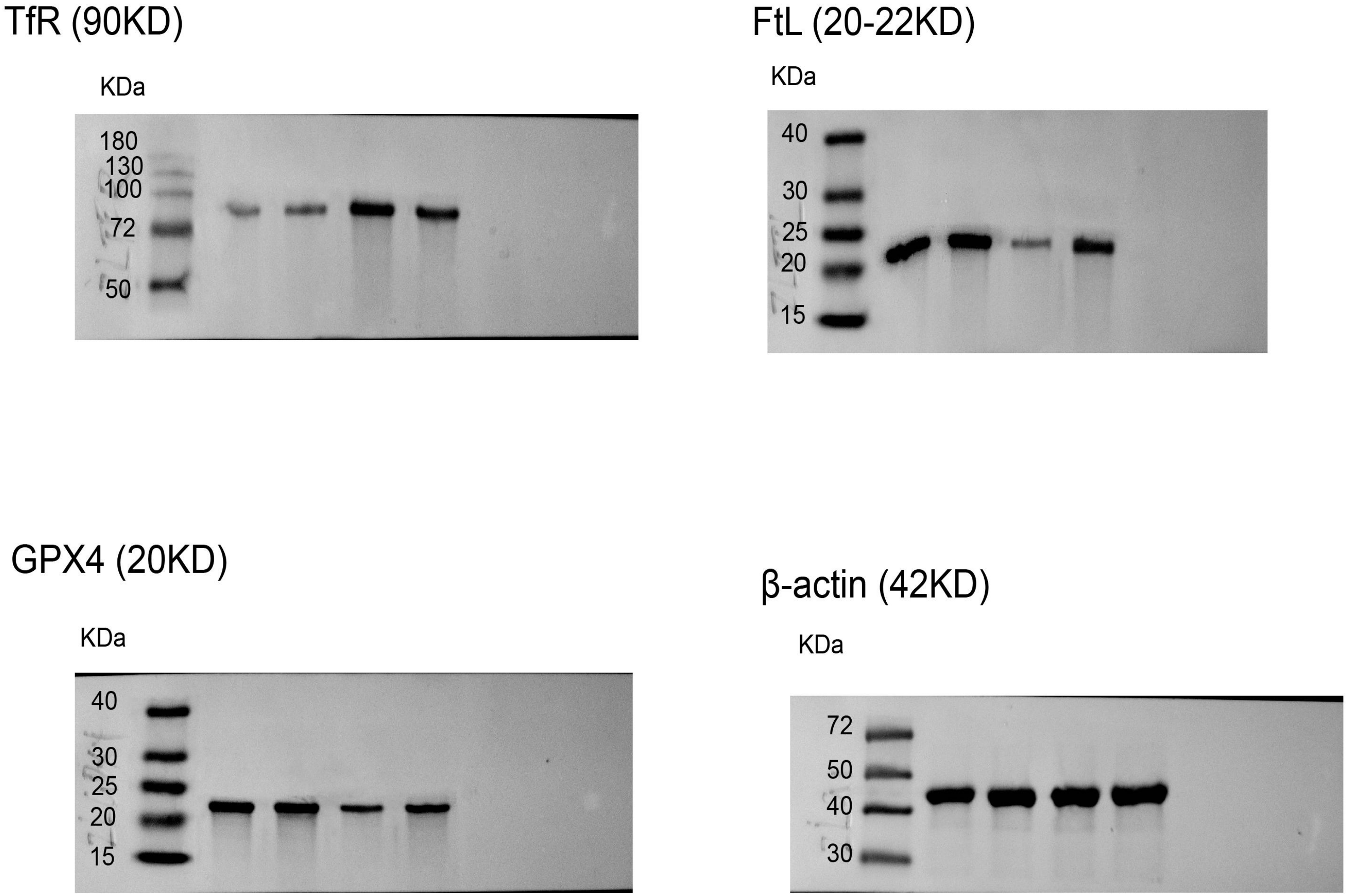

Original Image for Fig 2D

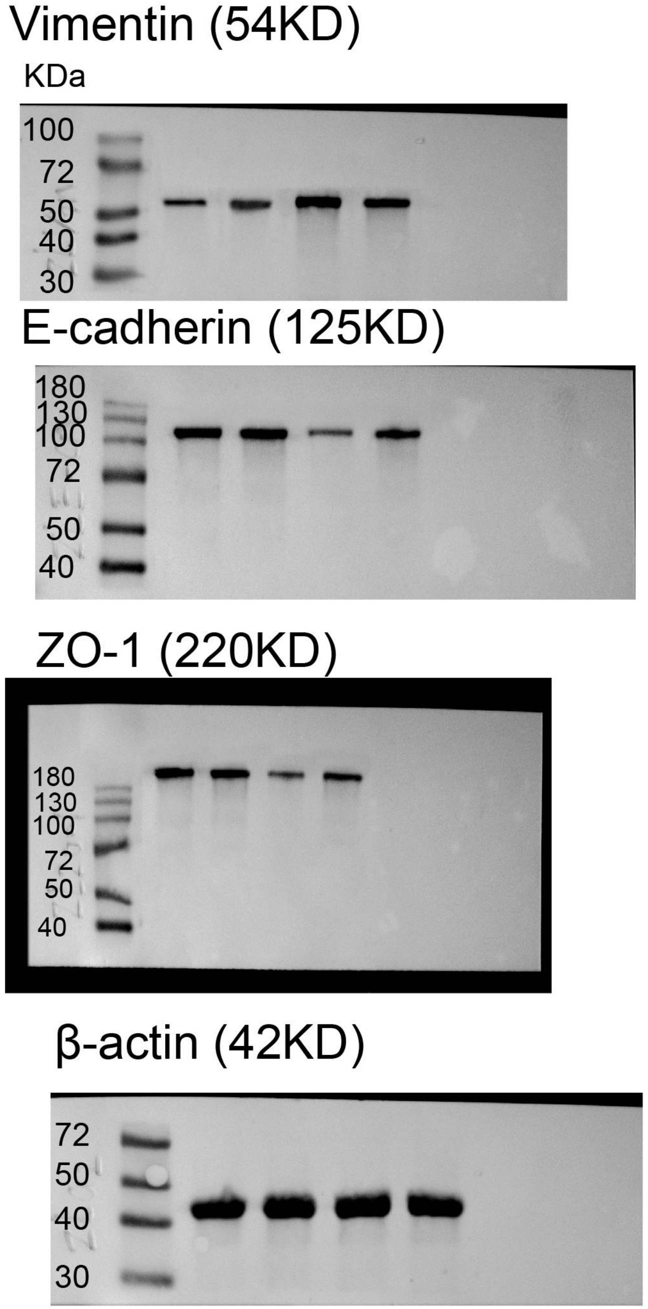

Original Image for Fig 3C

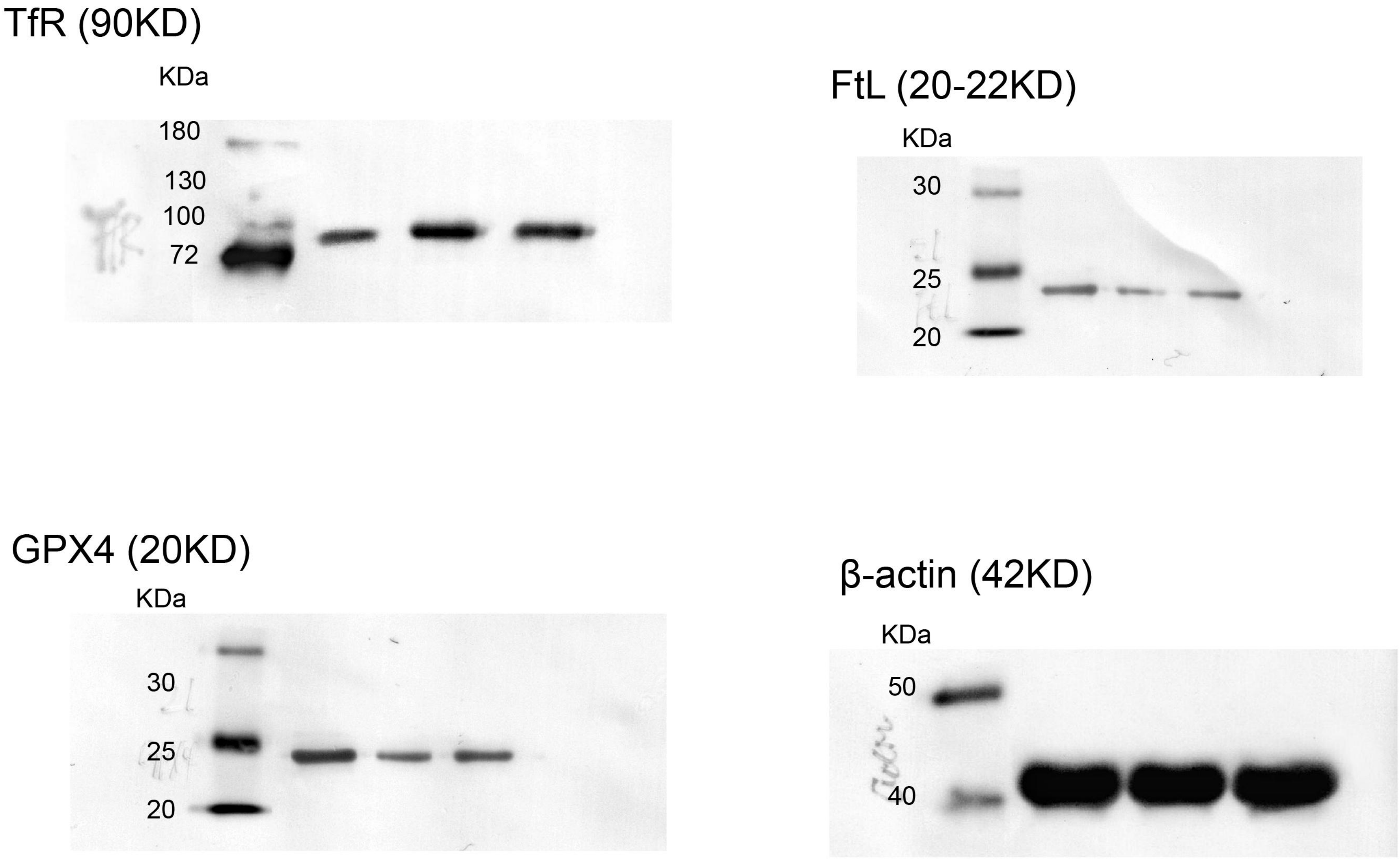

Original Image for Fig 4D

Vimentin (54KD)

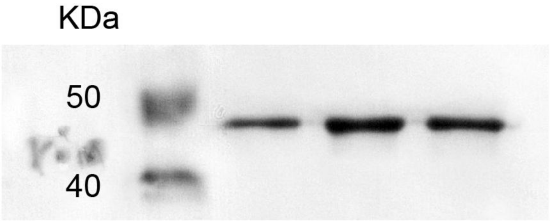

E-cadherin (125KD)

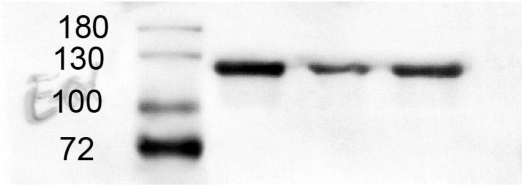

ZO-1 (220KD)

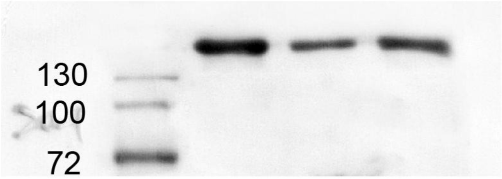

β-actin (42KD)

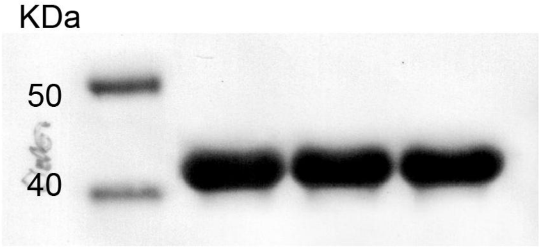

Original Image for Fig 4E

PTGS2 (69KD)

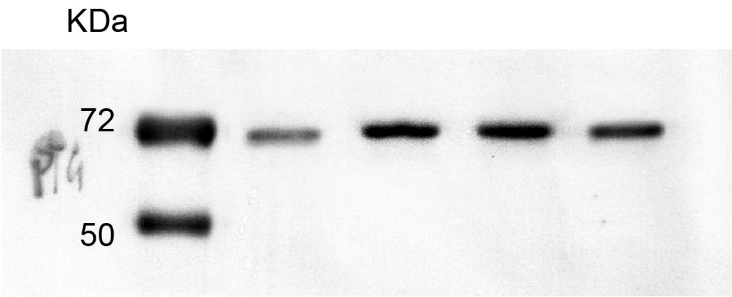

β-actin (42KD)

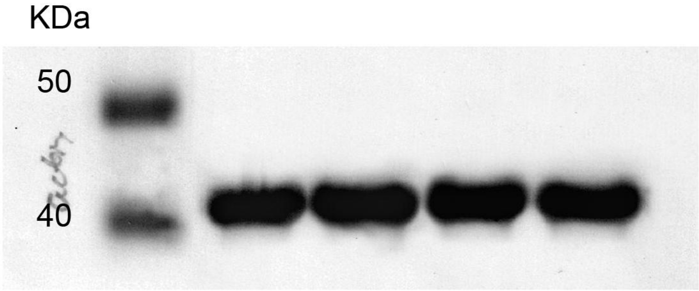

Original Image for Fig 5C

TfR (90KD)

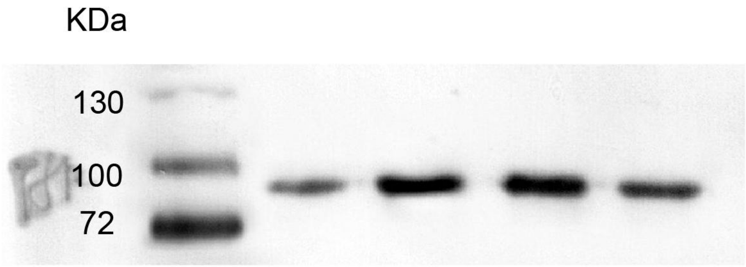

FtL (20-22KD)

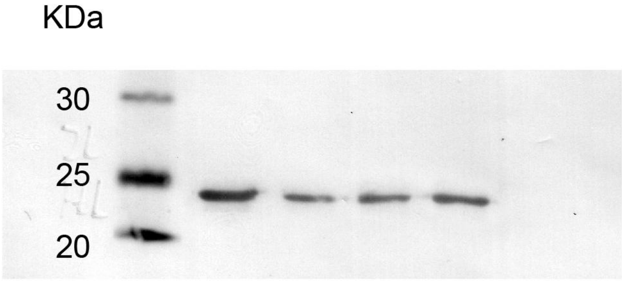

GPX4 (20KD)

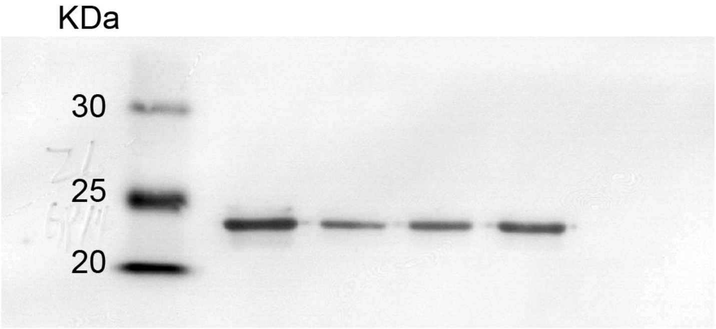

β-actin (42KD)

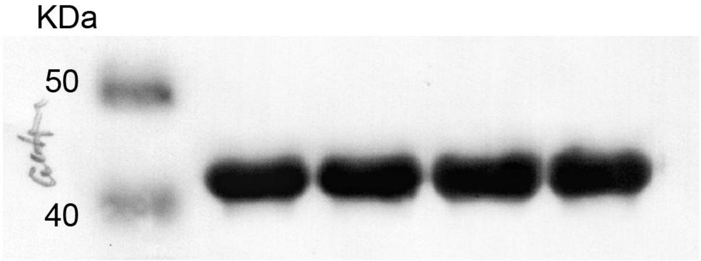

Original Image for Fig 5F

PTGS2 (69KD)

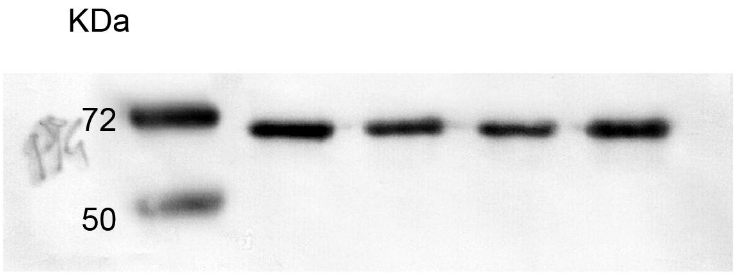

β-actin (45KD)

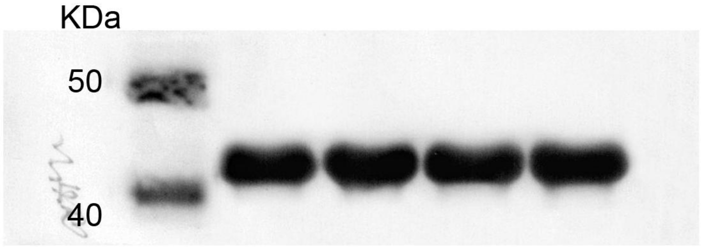

Original Image for Fig 6C

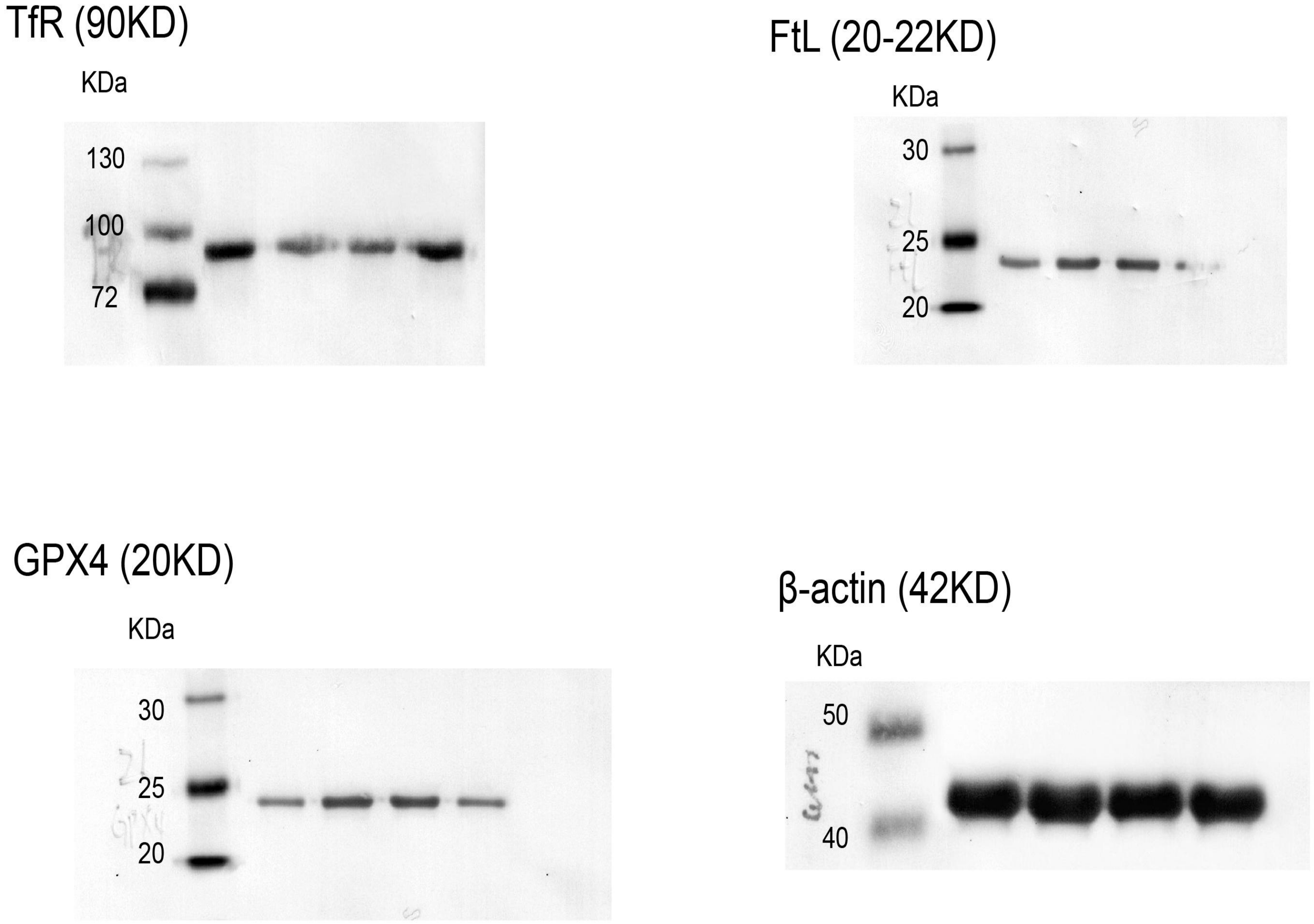

Original Image for Fig 6F

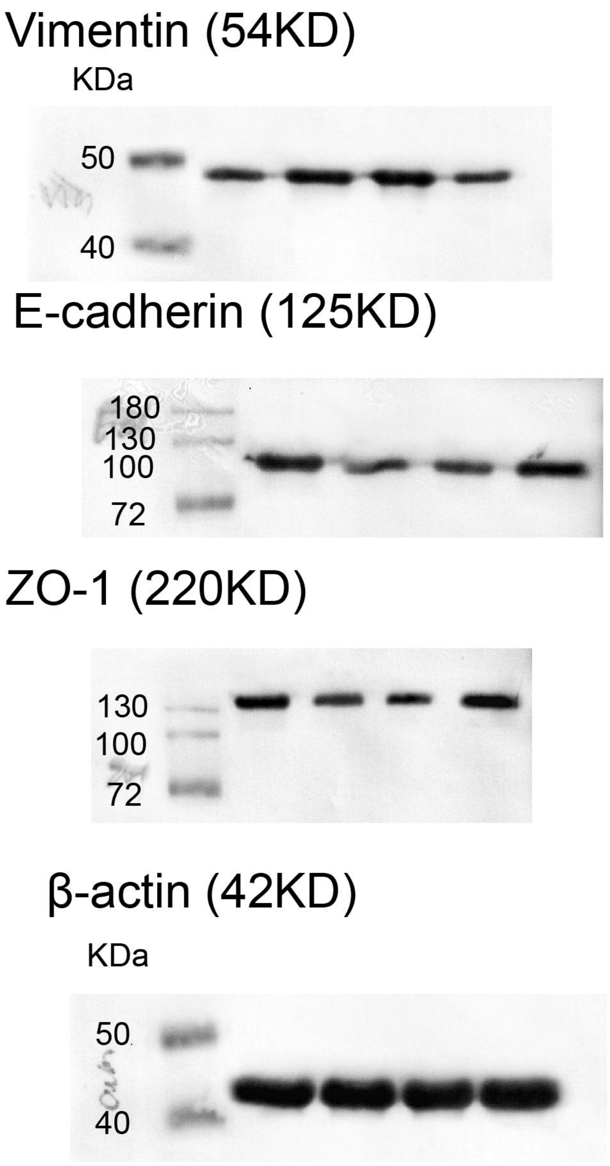

Original Image for Fig 7B

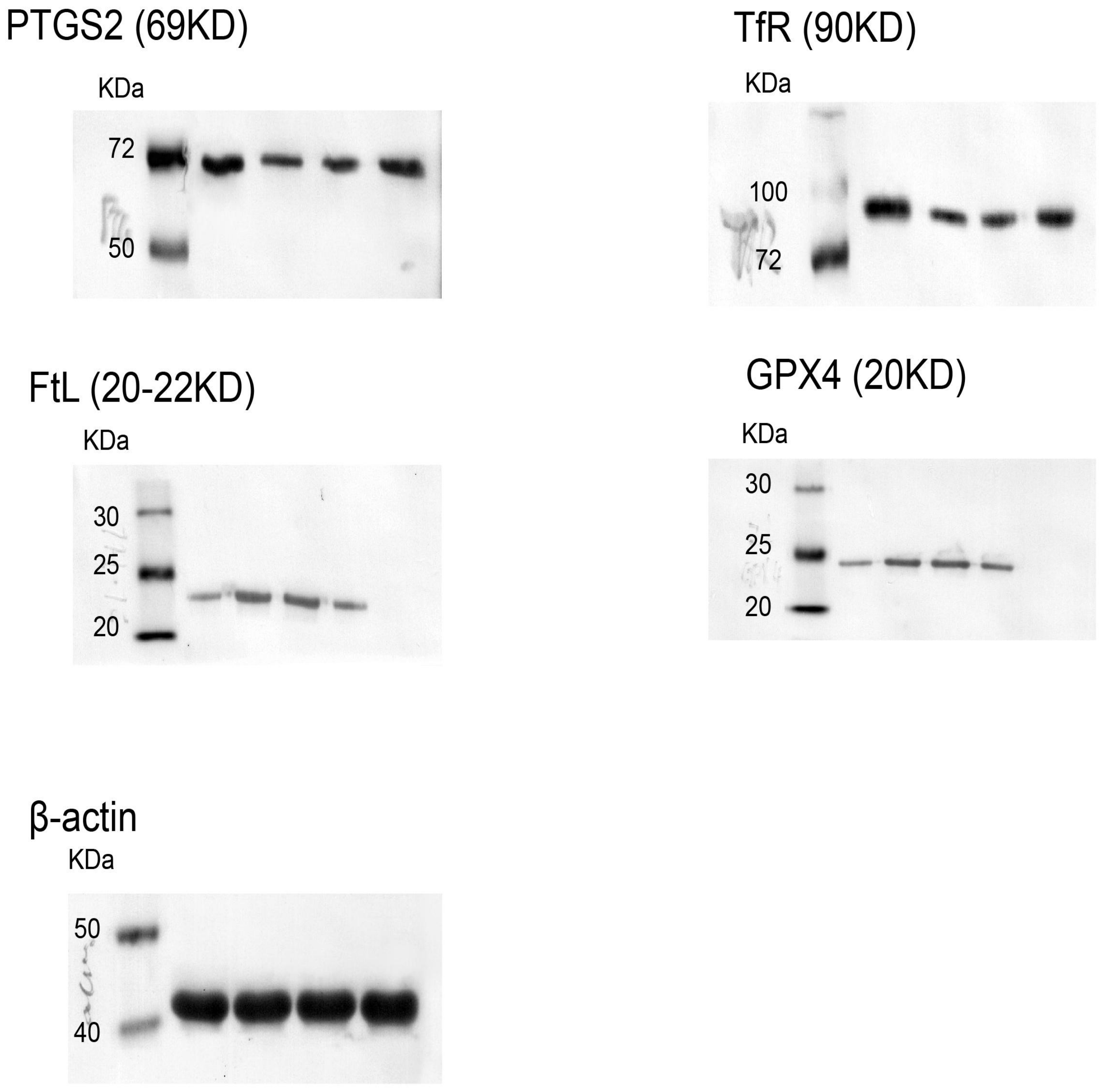

Original Image for Fig 8F

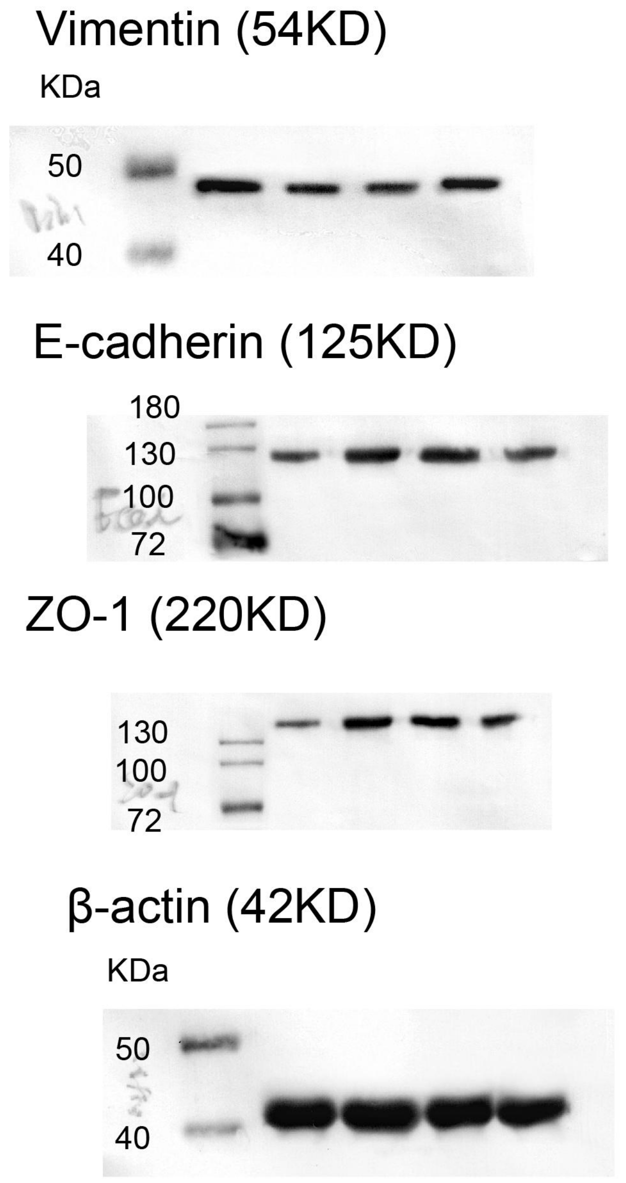

Original Image for Fig 8G

CD9 (23-30KD)

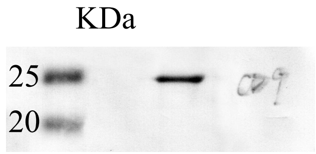

CD63 (28-35KD)

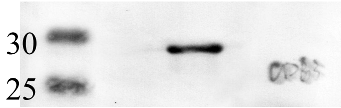

TSG101 (46KD)

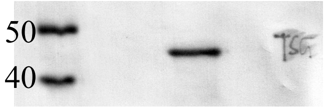

HSP90B1 (100KD)

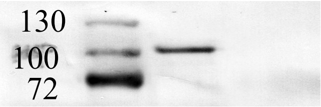

original image for Fig S1C
